# Solvent Fractionation and LC-MS Profiling, Antioxidant Properties, and α-Glucosidase Inhibitory Activity of *Bombyx batryticatus*

**DOI:** 10.3390/molecules30051021

**Published:** 2025-02-23

**Authors:** Guanhui Liu, Jingni Tang, Jie Tu, Xijie Guo

**Affiliations:** 1 College of Biotechnology, Jiangsu University of Science and Technology, Zhenjiang 212100, China; liuguanhui@just.edu.cn (G.L.); tujie@just.edu.cn (J.T.); 2 School of Grain Science and Technology, Jiangsu University of Science and Technology, Zhenjiang 212100, China; 212241821113@stu.just.edu.cn

**Keywords:** *Bombyx batryticatus*, antioxidant activity, α-glucosidase inhibition, *Beauveria bassiana*, extraction, metabolite profiling, composition analysis

## Abstract

*Bombyx batryticatus* is the dried body of silkworm (*Bombyx mori* Linnaeus) larvae infected with *Beauveria bassiana*. It is widely used in traditional Chinese medicine for treating convulsions, epilepsy, and hyperglycemia. In this study, *Bombyx batryticatus* and its extract were prepared. The total reducing power, hydroxyl radical scavenging and superoxide anion radical scavenging activities, as well as the α-glucosidase inhibitory activity of *Bombyx batryticatus* extract were superior to those of normal silkworm larvae extract. Among them, the IC_50_ value of *Bombyx batryticatus* extract for α-glucosidase was 5.76 mg/mL, while that of normal silkworm larvae extract was 7.0 mg/mL. Untargeted metabolomic analysis was employed to compare the material composition of normal silkworm larvae and *Bombyx batryticatus*. The results revealed 101 metabolic differences between the two groups, including a significant increase in fatty acids and their derivatives in the *Bombyx batryticatus* extract. Further separation and purification of the *Bombyx batryticatus* extract were performed using solvents of varying polarity. The chloroform fraction exhibited the highest inhibitory activity against α-glucosidase, with an IC_50_ value of 0.217 mg/mL. LC-MS further identified compounds in the chloroform fraction, suggesting that those alkaloids, fatty acids, and their derivatives may be responsible for its strong α-glucosidase inhibitory activity. This study elucidates the material basis underlying the pharmacological effects of *Bombyx batryticatus*, particularly its hypoglycemic components, thereby providing critical experimental support for its future development and application in medicine.

## 1. Introduction

In recent years, the rapid development of the global economy has led to significant changes in diet and lifestyle, contributing to a sharp rise in the global incidence of diabetes. Diabetes mellitus (DM) is a chronic metabolic disease primarily characterized by hyperglycemia, which results from either defective insulin secretion or insulin resistance [1]. According to the International Diabetes Federation (2021), the number of adults with diabetes worldwide reached 537 million in 2021, and this number is expected to rise to 783 million in 2045. Diabetes is often associated with hyperlipidemia, which significantly increases the risk of cardiovascular disease, neurodegenerative disease, kidney injury, and cancer [2]. Therefore, diabetes has become a critical global public health issue that requires urgent attention and management.

The search for anti-diabetic drugs derived from local natural resources, especially traditional drugs, has been pursued in many regions around the world, yielding achieved promising results. These natural resources typically contain bioactive substances with good antioxidant capacity, which can exert beneficial effects by neutralizing reactive oxygen species (ROS), enhancing endogenous antioxidant defense, and regulating key signaling pathways involved in glucose and lipid metabolism [3,4]. Ighodaro et al. [5] reported that extracts of *Sapium ellipticum* Pax leaf from Africa were rich in antioxidant compounds such as amentoflavone, lupeol, and luteolin-7-*O*-glucoside and exhibited hypoglycemic effects in rats. *Acalypha hispida* leaves from Indonesia, rich in antioxidant polyphenols, also showed hypoglycemic effects in rats [6]. Additionally, these natural resources may contain compounds that inhibit the activity of α-glucosidase. α-Glucosidase is one of the target enzymes of clinical hypoglycemic drugs and can delay the digestion and absorption of carbohydrates, thereby lowering postprandial blood glucose levels. For example, 1-deoxynojirimycin (1-DNJ) was screened from mulberry leaves due to its strong α-glucosidase inhibitory activity, which is now recognized as a hypoglycemic functional agent. Therefore, screening for anti-diabetic drugs from natural resources based on antioxidant and α-glucosidase inhibition activity has become a promising strategy [7,8]. These traditional medicines or natural resources are becoming supplements or substitutes for daily hypoglycemic drugs and also an important source of developing new anti-diabetes drugs.

*Bombyx batryticatus* (*B. batryticatus*), also known as stiff silkworm, is a traditional medicine used in China, South Korea, and Japan. According to the *Compendium of Materia Medica* from the Ming Dynasty in China, *B. batryticatus* was used as a remedy to treat conditions such as convulsions, epilepsy, sore throats, persistent headaches, and traumatic infections. *B. batryticatus* is the dried body of the silkworm (*Bombyx mori* Linnaeus) that forms following infection with *Beauveria bassiana* (*B. bassiana*) in the fourth–fifth instar larvae stages of the silkworm. *B. bassiana* secretes various hydrolytic enzymes, including lipases, proteases, and chitinases, which actively invade the silkworm’s body and proliferate within the larvae’s body cavities. Through competition for nutrients, interference with metabolism, and the secretion of toxins, the infection ultimately results in the death of the host [9]. Once the body of the dead silkworm is covered with white mycelium and conidia, the body is dried and processed into the final *B. batryticatus* product. Silkworm larvae are recognized as unique anti-diabetic agents and hypoglycemic health foods in Asia [10,11]. It is widely believed that silkworms exhibit significant hypoglycemic activity through the enrichment of 1-DNJ, flavonoids, and phenolic compounds through the consumption of mulberry leaves [10,12]. Compared to silkworms, the phenolic compound and fatty acid profiles of *B. batryticatus* have undergone alterations [13], contributing to its demonstrated anticonvulsant, antithrombotic, antiepileptic, and neuroprotective effects [14,15,16]. Recently, in the clinical applications of traditional Chinese medicine, *B. batryticatus* has been regarded as a valuable ingredient in the treatment of diabetes, dyslipidemia, and nephrotic syndrome. However, the material basis underlying the hypoglycemic effect of *B. batryticatus* remains unclear. Therefore, this study evaluated the anti-diabetic potential of *B. batryticatus* by assessing its antioxidant capacity and α-glucosidase inhibitory activity and further attempted to identify the bioactive components contributing to this potential. The results of this study will provide experimental evidence for the practical application of *B. batryticatus* in hypoglycemic agents and support its future use in preventing and treating diseases associated with oxidative stress, including anti-aging and neuroprotection.

## 2. Results and Discussion

### 2.1. Morphological Changes During Silkworm Infection with B. bassiana

**Figure 1:**
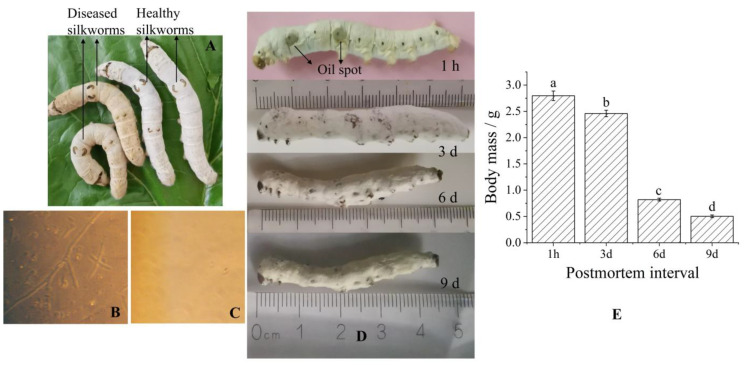
Disease symptoms and morphological changes in larvae infected with *B. bassiana*: (**A**) Body posture of silkworms 3 days after inoculation with *B. bassiana*. (**B**) Blastospores and hyphae of *B. bassiana* in the hemolymph of diseased larvae (×400). (**C**) Hemolymph of healthy larvae (×400). (**D**) Morphological changes in dead larvae. (**E**) Weight changes in dead larvae. Different lowercase letters (a–d) indicate significant differences among the means (*p* < 0.05).

Healthy fifth instar silkworm larvae were inoculated with the conidiospores of *B. bassiana*, and, by the third day, the infected larvae exhibited a loss of appetite and slow movement. The surface color of the infected larvae darkened compared to that of healthy larvae, and oil spots gradually appeared on the epidermis (Figure 1A). The observation of hemolymph samples under an inverted microscope (CKX-31, Olympus Co., Ltd., Tokyo, Japan) revealed the presence of hyphae and blastospores in the hemolymph of the infected larvae (Figure 1B), while the hemolymph of healthy larvae remained clear (Figure 1C). These findings indicate that the conidiospores of *B. bassiana* successfully infected and formed hyphae in the hemolymph of the infected larvae, and the rapid growth of the hyphae consumed water and nutrients in the hemolymph, which is a key factor contributing to the color changes in the epidermis [17]. On the fifth day of infection, a large number of infected larvae died, and the deceased larvae were collected and kept at room temperature for further observation (Figure 1D). Initially, the bodies of the just deceased larvae were soft with oil stains on the surface. Over time, the bodies gradually stiffened, and mycelia emerged from the cuticle junctions. On the third day after death, the surface of the stiffened silkworm was completely covered by white mycelium. Spores of *B. bassiana* then gradually formed. As storage time progressed, the silkworm bodies gradually became dry and hard, and their weight continuously decreased until the ninth day, reaching 18% of the original fresh weight (Figure 1E). Eventually, the bodies became rigid structures with a grayish-white or grayish-yellow surface, exhibiting a curved and wrinkled appearance.

### 2.2. The Antioxidant Activity of B. batryticatus Extract

**Figure 2:**
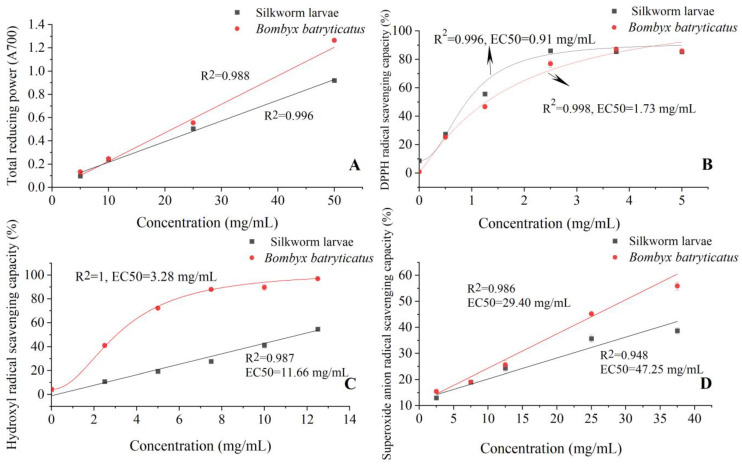
Total reducing power (**A**), DPPH radical scavenging ability (**B**), hydroxyl radical scavenging ability (**C**), and superoxide anion radical scavenging ability (**D**) of *B. batryticatus* extract.

To comprehensively evaluate the antioxidant activities of *B. batryticatus* extracts (80% methanol), various in vitro testing systems were used in this study (Figure 2). The value of total reducing power indicates the antioxidant potential to participate in free radical reactions and combine with free radicals to generate stable substances. As the extract concentration increased, the total reducing power of *B. batryticatus* also increased. The total reducing power reached 0.556 when the extract concentration was 25 mg/mL. Compared with the extract from healthy larvae, the total reducing power of *B. batryticatus* extract was 10% higher. At a concentration of 50 mg/mL, the total reducing power of *B. batryticatus* extract was 38% higher than that of healthy larvae extract (Figure 2A). 2,2-Diphenyl-1-picrylhydrazyl (DPPH) is a substance containing stable nitrogen radicals, and its ethanol solution is dark purple with a strong absorption peak at 517 nm. Free radical scavengers donate hydrogen atoms to react with DPPH free radicals, reducing them to colorless 2,2-diphenyl-1-pyridylhydrazine, thereby decreasing the absorbance value at 517 nm. The EC_50_ (half maximal effective concentration) of *B. batryticatus* extract was 1.73 mg/mL, while that of the healthy larvae extract was 0.91 mg/mL (Figure 2B). Hydroxyl radicals and superoxide anions are both highly harmful reactive oxygen species to living organisms. In the human body, they attack biomolecules, leading to diseases caused by oxidative stress. The inhibitory ability of *B. batryticatus* extract on hydroxyl radicals and superoxide anions was much higher than that of healthy larvae extract. The EC_50_ of *B. batryticatus* extract for hydroxyl radials was 3.28 mg/mL, while the value for the healthy larvae extract was 11.66 mg/mL. The EC_50_ of *B. batryticatus* extract for superoxide anions was 29.40 mg/mL, while that of the healthy larvae extract was 47.25 mg/mL (Figure 2C,D).

It was reported that the 80% methanol extract of freeze-dried powder from the fifth instar silkworm larvae had a high DPPH radical scavenging capacity (137.22 μM of α-tocopherol/g), with the total reducing power of 19.55 μM of Fe(II)/g [18]. The DPPH free radical scavenging ability of silkworm larvae powder from different strains ranges from 20.97% to 58.96%, and the Ferric ion reducing antioxidant power (FRAP) ranges from 1.52 to 2.03 mg Fe(II)/g [19]. The antioxidant capacity of silkworm larvae powder is primarily attributed to the presence of flavonoids [19]. Additionally, silkworm larvae contain unsaturated fatty acids [20] such as oleic acid, linolenic acid, arachidonic acid, docosahexaenoic acid, and bioactive polysaccharides [21], which is also the reason why silkworm powder has antioxidant capacity.

In this study, the antioxidant capacity of *B. batryticatus* extract was higher than that of healthy larvae extract. This may be attributed to the secretion of hydrolytic enzymes by *B. bassiana* in silkworm larvae, which produces more free fatty acids, free amino acids, and peptides. Cermeño reported that the FRAP values of silkworm pupae increased after enzymatic hydrolysis [22]. Bae et al. treated silkworm powder with proteolytic enzymes, resulting in an increase in the content of free amino acids and flavonoid, and an increase in the DPPH free radicals scavenging abilities [23]. On the other hand, *B. bassiana* is an entomopathogenic fungus. The interaction between *B. bassiana* and its host produces a series of bioactive compounds, including benzene ring derivatives (e.g., salicylaldehyde), terpenes (e.g., myrcene), benzoquinones (e.g., oosporein), unsaturated fatty acids, amides, lactones, and antimicrobial peptides [24]. These compounds interact with free amino acids, fatty acids, carbohydrates, and other biomolecules to form more complex secondary metabolites. Chemical structure analyses have shown that compounds containing unsaturated bonds and phenyl groups typically exhibit antioxidant activity [25,26]. These compounds can eliminate free radicals, regulate antioxidant enzyme activity, or reduce inflammatory mediators, thereby mitigating oxidative stress. Additionally, the biotransformation of flavonoids may also influence the antioxidant capacity of the extracts. It has been reported that *B. bassiana* can transform quercetin into quercetin-7-O-β-d-4-O-methylglucoside and can also transform kaempferol into kaempferol-7-O-β-d-4-O-methylglucoside [13]. The derivatization of quercetin (and kaempferol) hydroxyl groups reduced the antioxidant activity of their derivatives [27]. The fermentation by *B. bassiana* produces quercetin 7-O-β-D-(4″-O-methyl) glucopyranoside, which can effectively protect lipid membranes against peroxidation while exhibiting slightly lower antioxidant activity than quercetin [28]. However, the combination of flavonoid glycosides with methyl and sugar groups increases the absorption of flavonoids, leading to an increase in their bioavailability and biological activity [29]. In summary, when healthy silkworm larvae were transformed into *B. batryticatus*, the metabolic profiles changed, leading to a significant increase in antioxidant components and enhanced antioxidant capacity. Abdel-Daim et al. reported that compounds with antioxidant capacity provide multiple health benefits in preventing and treating disease [30]. Compared with healthy silkworm larvae, *B. batryticatus* demonstrated potential for more effective oxidative stress resistance and the prevention of metabolic and neurodegenerative disorders, as revealed by our findings.

### 2.3. The Inhibitory Activities of B. batryticatus Extract on α-Glucosidase and Pancreatic Lipase

**Figure 3:**
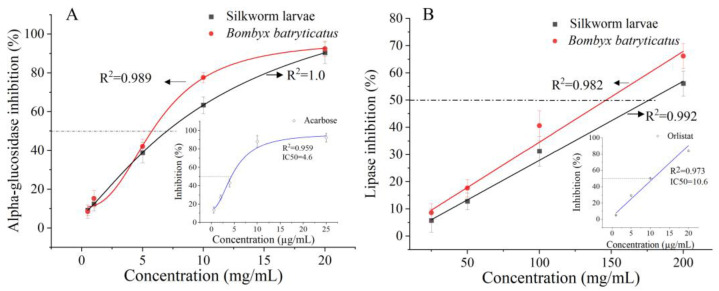
The inhibitory activities of *B. batryticatus* extract on α-glucosidase (**A**) and pancreatic lipase (**B**).

α-Glucosidase is a key enzyme that regulates postprandial blood glucose levels. Inhibiting this enzyme reduces carbohydrate absorption in the upper small intestine, delaying glucose uptake and thereby lowering postprandial blood glucose levels. Therefore, α-glucosidase inhibition is a therapeutic target for diabetes treatment. Extracts from healthy silkworm larvae were used as control samples in this study, while the clinical drugs acarbose (an α-glucosidase inhibitor) and orlistat (a lipase inhibitor) served as positive controls for in vitro hypoglycemic and lipid-lowering effects. As shown in Figure 3A, acarbose, *B. batryticatus* extract, and healthy silkworm larvae extract all showed dose-dependent inhibitory effects on α-glucosidase in vitro. As the concentration of *B. batryticatus* extract increased, α-glucosidase inhibition rose rapidly before gradually stabilizing. The IC_50_ (the concentration of the inhibitor required to provide 50% inhibitory activity) value of *B. batryticatus* extract for α-glucosidase was 5.76 mg/mL, which was lower than that of healthy silkworm larvae extract (7.0 mg/mL). In the same enzymatic reaction system, the IC_50_ value of acarbose for α-glucosidase was 4.9 μg/mL. The results indicate that the extract of *B. batryticatus* exhibits stronger inhibitory activity against α-glucosidase. The highest levels of 1-deoxynojirimycin (1-DNJ), flavonoids, phenolics, and other compounds were found in Thai silkworm races on the 3rd day of the fifth instar, with an IC_50_ value of 2 mg (silkworm powder)/mL for α-glucosidase [10]. So, the further screening of different silkworm strains remains highly significant for the preparation of *B. batryticatus* with medicinal value.

Pancreatic lipase plays a crucial role in triglyceride digestion by converting dietary fats into glycerol and fatty acids. Therefore, inhibiting the activity of pancreatic lipase in the intestine can reduce fat digestion and absorption, thereby lowering blood lipid levels. As shown in Figure 3B, pancreatic lipase inhibition was positively correlated with the concentration of *B. batryticatus* extract or healthy larvae extract. The IC_50_ value of *B. batryticatus* extract for pancreatic lipase was 147.9 mg/mL, which was slightly better than that of the healthy larvae extract (176 mg/mL). However, the inhibitory activities of the extracts were both significantly lower than that of Osalide (IC_50_ = 10.6 µg/mL).

Natural materials rich in flavonoids have been reported to exhibit good inhibitory activity against α-glucosidase and pancreatic lipase [31]. Hasnat H. concluded that flavonoids, such as quercetin and kaempferol, possess antioxidant, antidiabetic, and lipid-lowering activities [32]. In most cases, flavonoid aglycones exhibit stronger antioxidant activity, lipid peroxidation inhibition, and other biological effects in vitro than glycosides, including α-glucosidase inhibition, α-amylase inhibition, anti-inflammatory activity, and the ability to inhibit advanced glycation end products (AGEs) [33].

Silkworm larvae are processed into health foods and drugs due to their ability to inhibit α-glucosidase activity [34], lower blood sugar levels [11], regulate liver lipid metabolism, delay aging, and enhance resistance to Parkinson’s disease [18,35]. In this study, the α-glucosidase and pancreatic lipase inhibitory activities of *B. batryticatus* extract were higher than those of healthy silkworm extract, indicating its potential to combat hyperglycemia and hyperlipidemia. During the infection process of *B. bassiana*, various hydrolytic enzymes are produced, including chitinase, lipases, and proteases [36]. The hydrolysis of the enzymes produces additional bioactive components. For example, proteases generate peptides and free amino acids, which may contribute to the enhanced α-glucosidase and pancreatic lipase inhibitory activities of *B. batryticatus* extract. In addition, *B. bassiana* has been reported to produce lactones, quinolines, piperidines and other secondary metabolites that inhibit α-glucosidase and lipase activity [37]. In recent years, these numerous compounds with such functional groups have been used in the treatment of diabetes and its complications due to their inhibition on α-glucosidase or dipeptidyl peptidase IV [38].

### 2.4. Nontargeted Analysis of Samples by UPLC-TQ-LIT-MS

**Figure 4:**
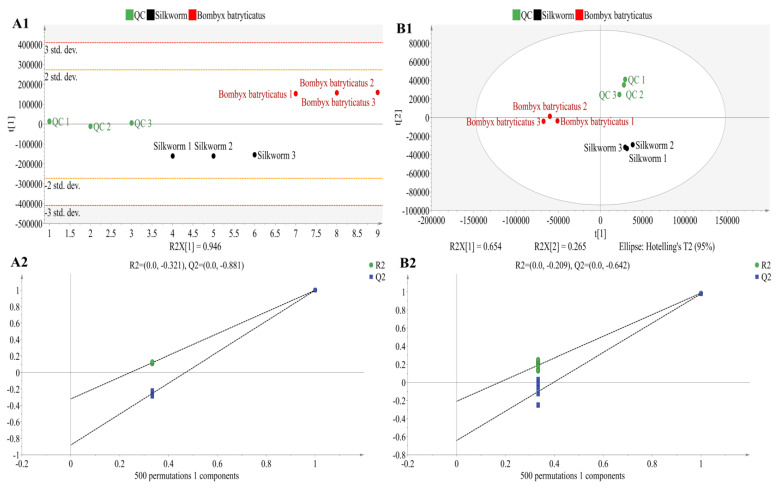
PCA scores plots (**A1**,**B1**) and OPLS-DA permutation test plots (**A2**,**B2**) in positive ion mode (**A1**,**A2**) and negative ion mode (**B1**,**B2**) between *B. batryticatus* and healthy larvae groups.

To investigate the chemical composition of *B. batryticatus*, the metabolic profiles of *B. batryticatus* and healthy silkworm larvae were compared. In the positive ion mode, 87 differential metabolites were detected, while 73 were identified in the negative ion mode. A principal component analysis (PCA) model was constructed using data from both ion modes as an unsupervised analytical method. As shown in Figure 4A1,B1, the *B. batryticatus* samples clustered distinctly from healthy larvae samples, with the quality control (QC) samples positioned between the two groups. The PCA plots revealed significant differences in the metabolic profiles between *B. batryticatus* and healthy larvae. In the positive ion mode, the PCA model yielded R^2^ and Q^2^ values of 0.942 and 0.89, respectively, while, in the negative ion mode, the values were 0.92 and 0.78, respectively. These results indicate that the PCA models in both ion modes have strong explanatory and predictive capabilities.

A supervised orthogonal partial least squares discriminant analysis (OPLS-DA) model was constructed using positive ion mode data, yielding an R^2^Y of 0.999 and a Q^2^ of 0.999. These values indicate that the model can effectively explain the differences between groups and demonstrates strong predictive capability. As shown in Figure 4A2, after 500 permutation tests, both R^2^ and Q^2^ values were lower than the original values. The intercept of the Q^2^ regression line was −0.852 (<0.05), confirming that the model did not overfit and was successfully constructed. Using negative ion mode data, another OPLS-DA model was constructed, yielding an R^2^Y of 0.865 and a Q^2^ of 0.978, with a difference of 0.113 (<0.3) between the two. Similarly, after 500 permutation tests, both R^2^ and Q^2^ values were lower than the original values, and the intercept of the regression line for Q^2^ was −0.624 (<0.05), confirming that the model did not overfit and that the diagnostic model was successfully constructed (Figure 4B2). These results indicated that the established OPLS-DA models perform well in interpreting and predicting data and effectively identified and discriminated between *B. batryticatus* and healthy larvae samples.

Variable importance in projection (VIP) reflects the contribution of each variable to the overall model fit and classification ability. According to a VIP score > 1.5 and *p* < 0.05, differential metabolites between healthy larvae and *B. batryticatus* were screened, as shown in Table 1. According to the VIP score, the differences are as follows: oleamide (VIP = 5.08, log_2_ fold change (FC) = 1.05), isoleucine (VIP = 4.57, log_2_FC = −2.52), 6-hydroxy-4-octadecenoic acid (VIP = 3.21, log_2_FC = −1.34), corchorifatty acid F (VIP = 3.20, log_2_FC = −1.09), palmitoyl ethanolamide (VIP = 3.17, log_2_FC = −3.32), ethyl palmitoleate (VIP = 2.98, log_2_FC = 6.84), stearoyl ethanolamide (VIP = 2.51, log_2_FC = −5.13), citric acid (VIP = 2.23, log_2_FC = 1.39), 13-hydroxy-9,11-octadecadienoic acid (VIP = 2.20, log_2_FC = −1.74), eicosapentaenoic acid (VIP = 1.62, log_2_FC = −2.55), and suberic acid (VIP = 1.60, log_2_FC = −2.46). Among them, eight metabolites were significantly upregulated in the *B. batryticatus*, primarily fatty acids and their derivatives. In contrast, ethyl palmitoleate was significantly downregulated. This is the result of the infection of silkworms by *B. bassiana*, which affected lipid metabolism.

Meanwhile, univariate statistical analysis (log_2_ FC > 1 or < −1, *p* < 0.05) was used to identify differential metabolites, as shown in Table 1. The results indicated a total of 101 metabolic differences between *B. batryticatus* extract and healthy larvae extract. Among them, 48 significantly upregulated metabolites were identified in healthy larvae extract, primarily esters, carboxylic acids, as well as some phenolic substances, purine substances, and carnitine substances. Certain phenolic compounds, such as gallic acid, may originate from mulberry leaves. The aforementioned compounds, for example, linolenic acid ethyl ester, cis-12-octadecenoic acid methyl ester, 1-linoleoyl glycerol, 10-hydroxy-2-decenoic acid, gallic acid, and acetyl-carnitine all exhibit antioxidant capacity in vitro or in vivo [39,40,41]. In *B. batryticatus* extract, 53 significantly upregulated metabolites were identified, primarily fatty acids and derivatives, as well as amino acids. Fatty acids are important energy storage substances in the body of silkworms. As shown in Table 1, the abundance of fatty acids and their derivatives in *B. batryticatus* extract significantly increased, including arachidonic acid, docosapentaenoic acid, eicosapentaenoic acid, palmitoleic acid, 13-hydroxy-9,11-octadecadienoic acid, 9-hydroxy-10,11-octadecadienoic acid, stearoyl ethanolamide, oleoyl ethanolamide, palmitoyl ethanolamide, dihomo-γ-Linolenoyl, ethanolamide, 2-naphthylamine. It has been reported that *B. bassiana*-infected silkworms showed an upregulation of fatty acid metabolites [13]. In addition, a similar phenomenon was observed in the infection of black fly larvae by *B. bassiana* [42]. These results suggest that *B. bassiana* infection triggers an energy-intensive immune response in silkworms. These unsaturated fatty acids have been reported to exhibit strong antioxidant capacity, α-glucosidase inhibition ability, and hypoglycemic potential [43,44]. Amide compounds also have good antioxidant capacity [45] and physiological functions, including anti-anxiety, anti-depressive, and sleep-regulating effects [46]. Especially, it exhibits strong α-glucosidase inhibitory activity and can reduce serum triglycerides (TGs) and total cholesterol (TC) [47]. In this study, as shown in Table 1, various amino acids and amino acid oxides were upregulated following *B. bassiana* infection, such as methionine sulfoxide (log_2_FC = −5.53), kynurenine (log_2_FC = −4.28), phenylalanine (log_2_FC = −3.24), arginine (log_2_FC = −2.63), isoleucine (log_2_FC = −2.52), N-acetyl-leucine (log_2_FC = −1.82), glutaric acid (log_2_FC = −1.77), serine (log_2_FC = −1.75), and ornithine (log_2_FC = −1.27). This phenomenon is due to proteases secreted by *B. bassiana*, which degrade host proteins, and the need for infected silkworms to accelerate the production of amino acids to cope with energy deficiency and synthesize antimicrobial peptides [48]. The similar phenomenon was reported in the studies of Xu (2015) and Huang (2009) [48,49]. Liu et al. confirmed that amino acids exhibit antioxidant capacity [50]. Additionally, some adenosine and glycosides are characteristic metabolites of *B. batryticatus*. In summary, *B. bassiana* infection significantly impacts the energy metabolism, nutrient metabolism, and immune defense mechanisms in the silkworm, leading to metabolic alterations in *B. batryticatus* and altering its antioxidant capacity, α-glucosidase, and lipase inhibitory capacity. It is worth noting that the compounds upregulated in *B. bassiana* (as shown in Table 1) contain some valuable bioactive molecules such as 13-hydroxy-9,11-octadecadienoic acid, trehalose, 3-carboxy-4-hydroxyphenyl-β- D-glucopyranoside, etc. 13-hydroxy-9,11-octadecadienoic acid has been reported to target breast cancer stem cells and inhibit their self-renewal and induce apoptosis [51]. Trehalose is associated with oxidative stress modulation, anti-aging, neuroprotection, and the regulation of cancer cell metabolism [52]. 3-Carboxy-4-hydroxyphenyl- β-D-glucopyranoside is a derivative of arbutin and therefore may have potential anti-inflammatory and pigment deposition prevention effects.

### 2.5. The Antioxidant Activity, Total Flavonoid Content, and α-Glucosidase Inhibitory Activity of Several Extracts of B. batryticatus

**Figure 5:**
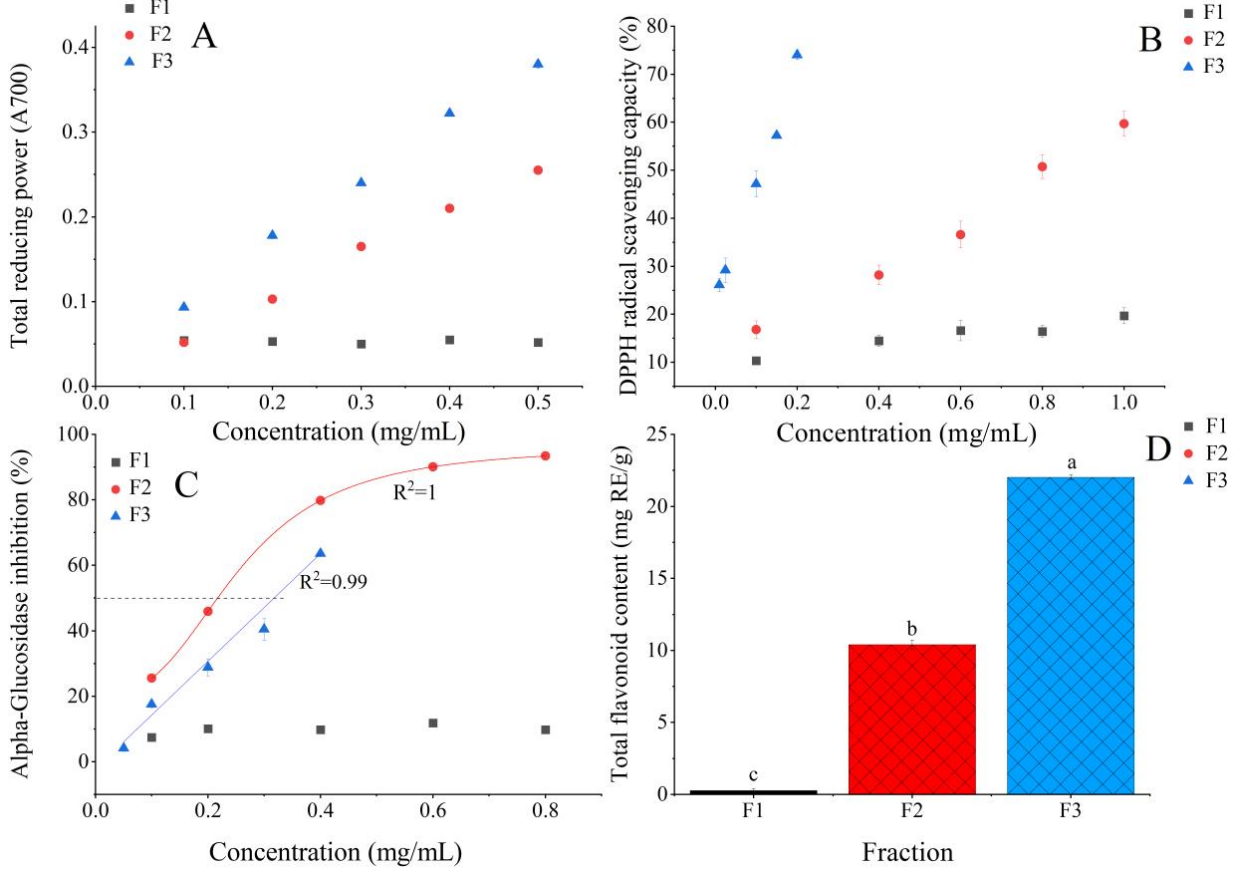
Total reducing power (**A**), DPPH radical scavenging activity (**B**), α-glucosidase inhibitory activity (**C**), and total flavonoid content (**D**) of the different fractions from *B. batryticatus* extract. Values marked by different letters (a, b, c) at the top of the bar chart are significantly different (*p* < 0.05).

By sequentially extracting *B. batryticatus* with solvents of increasing polarity, the chemical constituents were selectively distributed among different extraction fractions based on their polarity. There were significant differences in the antioxidant capacity (total reducing power shown as Figure 5A, DPPH radical scavenging capacity shown as Figure 5B) of the three polar fractions of the *B. batryticatus* extract, which were as follows: ethyl acetate fraction concentrate (F3) > chloroform fraction concentrate (F2) > petroleum ether fraction concentrate (F1). This result was attributed to the composition and concentration of antioxidant compounds in extracts from different polar solvents. Notably, although F2 exhibited lower antioxidant capacity than F3, it showed stronger inhibitory activity against α-glucosidase (IC_50_ = 0.217 mg/mL) (shown in Figure 5C). In accordance with the principles of polarity and solvent affinity, flavonoids were mainly concentrated in the ethyl acetate fraction, followed by the chloroform fraction (shown in Figure 5D).

### 2.6. Component Profiling of Chloroform Extract Concentrate (F2) by LC-MS

To identify the bioactive compounds in F2, LC-MS analysis was performed on the metabolites of the chloroform extract of *B. batryticatus*. The identified mass spectrometry data are shown in Table S1 (Supplementary Material). Since the peak area reflects the content of the corresponding substance in the sample, compounds with peak areas in the top 20 of F2 were prioritized for analysis. These compounds were classified and compared, and the results are shown in Table 2. Among these compounds, unsaturated fatty acids, phenolic acids, and flavonoids all exhibit good antioxidant activity. According to the chemical structure, threo-3-phenylserine with benzene rings, some ketones, and amides with unsaturated bonds also have the potential for antioxidant activity.

As shown in Table 2, many heterocyclic compounds containing oxygen or nitrogen were present in the chloroform composition. Substances with this chemical structure often have enzyme inhibitory activity, which has anti-inflammatory, hypoglycemic, and neuroprotective effects in vivo. F2-18 contains a fused structure consisting of furan and indole, which is an analog of benzofuran. Many compounds with a benzofuran structure have activities against diabetes, tumors, hyperlipidemia, convulsions, and Alzheimer’s disease [53]. It has been reported that spiroindolone analogs exhibited excellent dual inhibitory activity against α-amylase and α-glucosidase [54]. Therefore, the compound in F2 has good potential for anti-diabetic effects and may possess other pharmacological activities. F2-15 is a derivative of coumarin. Substances with a benzopyran structure (coumarin scaffolds) are very important in pharmaceutical chemistry, possessing antibacterial, antioxidant, and anti-cancer properties, and also have the potential to develop potent anti-diabetes drugs due to their α-glucosidase inhibitory activity [55]. F2-14 is a biologically active compound with a pyrrolopyrazine skeleton. Substances with this structure often exhibit anti-inflammatory, antiviral, antidepressant, cardiovascular protective, antifungal, antioxidant, antitumor, and kinase inhibitory properties [56]. Similarly, F2-17 is a biologically active compound with a pyrroloquinoline ring skeleton. Substances with this structure often exhibit neuroprotective and cardiovascular protective effects [57]. Moreover, F2-14 and F2-17 are both nitrogen-containing alkaloids. Therefore, they may also have the potential to inhibit α-glucosidase activity [58]. F2-16 contains a lactone group and naphthalene, so it may have pharmacological value.

In addition, the α-glucosidase inhibitory activity of quercetin, fatty acids, and their amide derivatives is well recognized. Salicylic acid and hydroxycinnamic acid both contain a phenolic hydroxyl structure and have been shown to exhibit α-glucosidase inhibitory activity [59,60]. It is noteworthy that, in this study, senkyunolide H was found in F2. Senkyunolide H is a natural phthalide compound with antioxidant properties, as well as anti-inflammatory and antithrombotic effects [61]. It also contains both phthalide and benzofuran characteristics in its structure. Senkyunolide has also been used in the treatment of Parkinson’s disease and thromboembolic disorders due to its ability to inhibit human monoamine oxidase B and thrombin [62,63]. Although there are currently no reports on the α-glucosidase inhibitory activity of senkyunolide, its unique chemical structure makes it a potential candidate for the treatment of diabetes, warranting further attention. In summary, due to the chemical structure of benzene rings, oxygen-containing rings, nitrogen-containing rings, and long fatty chains, these compounds are more enriched in F2 than in F3. Their synergistic effect provides the F2 with antioxidant capacity and strong α-glucosidase inhibitory activity.

## 3. Materials and Methods

### 3.1. Chemicals and Reagents

Analytical grade reagents for solvent extraction, total flavonoids, and antioxidant capacity determination experiments were purchased from Sinopharm Chemical Reagent Co., Ltd. (Shanghai, China). α-Glucosidase (activity: 50 U/mg, CAS: 9001-42-7) and pancreatic lipase (activity: 30 U/mg, CAS: 9001-62-1) were purchased from Yuanye Biotechnology Co., Ltd. (Shanghai, China). Acarbose was purchased from Bayer Biotechnology (China) Co., Ltd. (Shanghai, China). Orlistat was purchased from Hunan Dinuo Pharmaceutical Co., Ltd. (Changsha, China). p-Nitrophenyl α-D-glucopyranoside (pNPG, ≥98.0%, CAS: 3767-28-0) was purchased from Shanghai Huicheng Biotechnology Co., Ltd. (Shanghai, China). p-Nitrophenyl palmitate (pNPP, ≥98.0%, CAS: 1492-30-4) was purchased from Shanghai Aladdin Biochemical Technology Co., Ltd. (Shanghai, China). Methanol (for LC-MS, CAS: 67-56-1) and acetonitrile (for UHPLC-MS, CAS: 75-05-8) were purchased from Thermo Fisher Scientific, Inc. (Waltham, MA, USA), and formic acid (for LC-MS, CAS: 64-18-6) was purchased from LiChropur™ (Darmstadt, Germany).

### 3.2. Preparation of B. batryticatus

The silkworms (Jingsong × Haoyue strains) and *B. bassiana* used in this study were provided by the Silkworm Pathology and Physiology Laboratory of the Chinese Academy of Agricultural Sciences, Zhenjiang, China. A suspension of 1 × 10^6^ conidia/mL was sprayed onto newly exuviated fifth-instar silkworm larvae. The silkworms were then fed normally and began to die on the fifth day. Dead larvae were collected and placed in a humid environment until their bodies were gradually covered with white mycelium and became stiff. The cadavers were freeze-dried on the ninth day post-mortem using an LGJ-12D freeze dryer (Sihuan Scientific Instrument (Tianjin) Technology Co., Ltd., Tianjing, China). Healthy larvae were freeze-dried on the fifth day of their fifth instar. The freeze-dried samples, with a moisture content of less than 5%, were ground using a grinder (RS-FS1411, Royalstar Electronic Appliance Co., Ltd., Hefei, China), passed through an 80-mesh sieve, and then stored in a desiccator. The rearing of silkworms and the preparation of *B. batryticatus* were both conducted at the Sericulture Research Institute of Jiangsu University of Science and Technology.

### 3.3. Sample Extraction and Preparation

**Figure 6:**
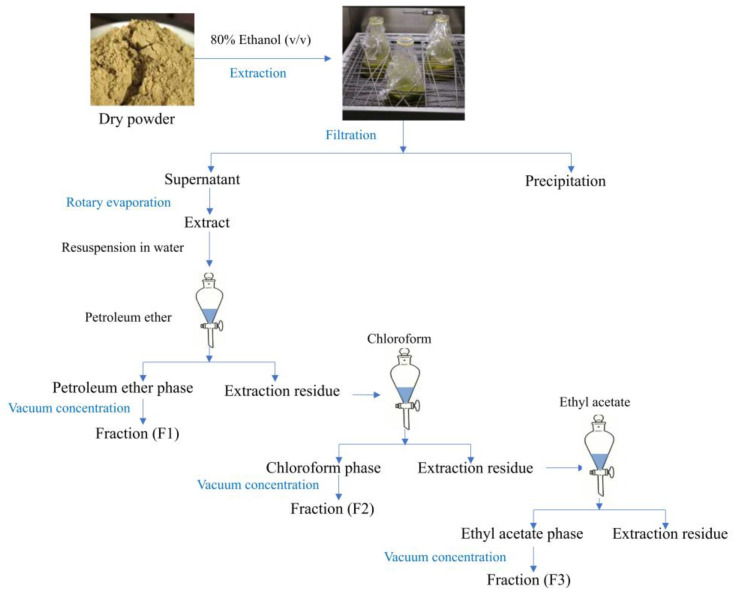
Fractional extraction process of *B. batryticatus*.

The freeze-dried sample (0.5 g) was extracted by vibration with 5 mL of 80% methanol for 60 min at room temperature. The extraction was repeated, and the extraction solutions were combined. The resulting solution was centrifuged at 6791× *g* for 10 min, and the supernatant was collected for testing antioxidant capacity, α-glucosidase inhibitory activity, pancreatic lipase inhibitory activity, and the analysis of extracts by UPLC-TQ-LIT-MS. As shown in Figure 6, the *B. batryticatus* powder (150.00 g) was repeatedly extracted with 80% ethanol until the extract became colorless. After filtration through Whatman No. 1 filter paper twice, the extract was concentrated using a rotary evaporator (RV 8, IKA Works GmbH & Co., Staufen, Germany) and then further concentrated in a vacuum dryer (DZF 6032, Shanghai Everone Precision Instruments Co., Ltd., Shanghai, China). The extract concentrate was suspended in a small amount of distilled water and successively partitioned with petroleum ether, chloroform, and ethyl acetate. The petroleum ether-concentrated extract (petroleum ether fraction, PEF: 23.341 g), chloroform-concentrated extract (chloroform fraction, CF: 0.258 g), and ethyl acetate-concentrated extract (ethyl acetate fraction, EAF: 0.096 g) were prepared. After dissolving each fraction in a trace amount of dimethyl sulfoxide, the solution was mixed with 80% methanol and used to measure antioxidant activity, total flavonoid content, and α-glucosidase inhibitory activity.

### 3.4. Total Flavonoid Determination

The total flavonoid content was determined using the colorimetric method [64]. It was calculated as rutin equivalents (REs) based on the standard curve (absorbance at 510 nm = 10.994x + 0.0173, R^2^ = 0.994), using five concentrations in methanol ranging from 0.05 to 0.4 mg/mL. The final results were expressed as milligrams of rutin equivalents per gram of dry extract weight (mg RE/g).

### 3.5. Evaluation of Antioxidant Capacity

#### 3.5.1. Total Reducing Power Assay

The total reducing power was determined using the method described by Tang [65]. Briefly, 1.00 mL of extraction solution with different concentrations (5, 10, 25, 50 mg/mL) was taken, followed by the addition of 2.50 mL of 0.2 M, pH 6.6 phosphate buffer, and, then, 2.50 mL of 1% potassium ferrocyanide (K_3_Fe(CN)_6_) was added. After 30 min in a water bath at 50 °C, 2.50 mL of 10% trichloroacetic acid was added, the mixture was mixed well, and, then, it was centrifuged at 4000 rpm for 10 min. In total, 2 mL of the centrifuged supernatant was mixed with 2 mL of distilled water and 0.4 mL of 0.1% ferric chloride, and colorimetric analysis was performed at 700 nm.

#### 3.5.2. DPPH Radical Scavenging Activity Assay

The DPPH radical scavenging activity was determined using the method described by Tang et al. [65]. Briefly, aliquots (150 μL) of extraction solutions at different concentrations (0, 1, 2.5, 5, 7.5, 10 mg/mL) were taken, followed by the addition of 250 μL of methanol DPPH solution (100 μmol/L). The mixture was left in the dark for 30 min at room temperature before measuring the absorbance (A_i_) at 517 nm. The absorbance measured using methanol solvent instead of the extraction solution was recorded as A_0_, and the absorbance measured using methanol solvent instead of the DPPH solution was recorded as A_j_. The result was calculated using the following formula:

(1) $$DPPH\;radical\;scavenging\;(\%)=(1-\frac{A_{i}-A_{j}}{A_{0}})\times 100,$$

the EC_50_ value is the quantity of antioxidant required to eliminate half of all free radicals.

#### 3.5.3. Hydroxyl Radical Scavenging Activity Assay

The hydroxyl radical scavenging activity was determined using the method described by Jiang et al. [66]. Briefly, aliquots (200 μL) of extraction solutions at different concentrations (0, 2.5, 5, 7.5, 10, 12.5 mg/mL) were taken, followed by the addition of 200 μL of 5 mmol/L FeSO_4_ and then 200 μL of 5 mmol/L salicylic acid ethanol solution. Subsequently, 200 μL of 5 mmol/L H_2_O_2_ was immediately added to initiate the reaction. After shaking well, the mixture was incubated at 37 °C for 30 min and then centrifuged at 7000 rpm for 3 min. The absorbance of the supernatant at 510 nm (A_510_) was measured. The absorbance measured by replacing the extraction solution with methanol was recorded as A_max_, while the absorbance obtained by replacing the corresponding H_2_O_2_ solution with distilled water was recorded as A_b_. The result was calculated using the following formula:

(2) $$Hydroxyl\;radical\;scavenging\;(\%)=\left(1-\frac{A_{510}-A_{b}}{A_{\max}}\right)\times 100$$

#### 3.5.4. Superoxide Radical Scavenging Activity Assay

The measurement method was slightly modified from that described by Tang et al. [65]. Briefly, aliquots (100 μL) of extraction solutions at different concentrations (2.5, 5, 7.5, 12.5, 25, 50 mg/mL) were taken, followed by the addition of 450 μL of 50 mmol/L Tris-HCl buffer (pH 8.2) and 400 μL of 25 mmol/L pyrogallol solution. The mixture was thoroughly mixed and reacted at 25 °C for 5 min. Then, 100 μL of 8 mmol/L HCl was added to terminate the reaction, the mixture was shaken well, left to stand for 3 min, and the absorbance (A_420_) was measured at 420 nm. The absorbance measured by replacing the extraction solution with methanol was recorded as A_c_, while the absorbance measured by using distilled water instead of the pyrogallol solution was recorded as A_d_. The result was calculated using the following formula:

(3) $$Superoxide\;radical\;scavenging\;(\%)=\left(1-\frac{A_{420}-A_{d}}{A_{c}}\right)\times 100$$

### 3.6. α-Glucosidase Inhibition Assay

The measurement method was slightly modified from that described by Tu et al. [67]. Briefly, the enzyme reaction system consisted of 200 µL of a 0.05 mol/L phosphate buffer (pH 6.8), 400 µL of a 0.2 U/mL α-glucosidase solution, 20 µL of extraction solution (0.5–20 mg/mL) or acarbose solution (0.5–25 µg/mL), and 40 µL of 0.02 mol/L substrate solution (pNPG). The reaction mixture was incubated at 37 °C for 20 min, and the reaction was terminated by adding 160 µL of 1 mol/L sodium carbonate solution. The absorbance of the mixed solution was measured at 405 nm. The experiment was repeated three times, and the average value was used to calculate the inhibitory activity and determine the IC_50_ value from the regression curve.

(4) $$\alpha -Glucosidase\;inhibitory\;(\%)=\left(1-\frac{A_{\mathrm{sample}}-A_{\mathrm{blank}}}{A_{\mathrm{test}}-A_{control}}\right)\times 100,$$

where A_sample_ is the absorbance value of a mixture of extraction solution, enzyme solution, and pNPG solution; A_blank_ is the absorbance value of a mixture of extraction solution, enzyme-free buffer solution, and pNPG solution; A_test_ is the absorbance value of a mixture of buffer solution, enzyme solution, and pNPG solution; and A_control_ is the absorbance value of a mixture of buffer solution and pNPG solution.

### 3.7. Pancreatic Lipase Inhibition Assay

The measurement method was slightly modified from that described by Aloo et al. [68]. Briefly, the enzyme reaction system consisted of 100 µL of 50 mmol/L Tris-HCl buffer (pH 7.5) containing sodium cholate and arabic gum, 40 µL of extraction solution (25–200 mg/mL) or orlistat solution (1–20 µg/mL), 100 µL of 10 mg/mL pancreatic lipase solution, and 160 µL of 16 mmol/L substrate solution (pNPP). After incubation at 37 °C for 20 min, the absorbance was measured at 405 nm, and the inhibitory activity was calculated using the following formula. The experiment was repeated three times, and the average value was used to create a regression curve and calculate the IC50 value.

(5) $$Pancreatic\;lipase\;inhibitory\;(\%)=\left(1-\frac{A_{\mathrm{sample}}-A_{\mathrm{blank}}}{A_{\mathrm{test}}-A_{control}}\right)\times 100,$$

where A_sample_ is the absorbance value of a mixture of an extraction solution, enzyme solution, and pNPP solution; A_blank_ is the absorbance value of a mixture of an extraction solution, enzyme-free buffer solution, and pNPP solution; A_test_ is the absorbance value of a mixture of a buffer solution, enzyme solution, and pNPP solution; and A_control_ is the absorbance value of a mixture of a buffer solution and pNPP solution.

### 3.8. UHPLC-TQ-LIT-MS Analysis

The detection procedure was conducted using an Orbitrap Exploris 240 high-resolution mass spectrometer (Thermo Scientific, Waltham, MA, USA), coupled with an ultra-performance liquid chromatography (UHPLC) system (Thermo Scientific, Waltham, MA, USA) and operated with Xcalibur data acquisition software (version 4.1, Thermo Scientific, USA). Data processing involved spectral library retrieval from MzCloud, ChemSpider, and Mass List databases, using Compound Discoverer software (version 3.2, Thermo Scientific, USA). Chromatographic separation was carried out on a Hypersil Gold C18 column (1.9 µm, 100 mm× 2.1 mm; Thermo Scientific, USA), with an injection volume of 3 µL and a column temperature of 50 °C. The mobile phase comprised solvent A (0.1% formic acid in acetonitrile) and solvent B (0.1% formic acid in aqueous solution). The column was eluted at a flow rate of 0.38 mL/min, following the gradient program: the initial conditions started at 98% solvent B, held for 0–0.5 min; from 0.5 to 1.5 min, solvent B was reduced from 98% to 60%; from 1.5 to 6.5 min, solvent B decreased from 60% to 20%; from 6.5 to 9.5 min, solvent B was reduced to 0%; from 9.5 to 13 min, solvent B was maintained at 0%; from 13 to 13.4 min, solvent B increased from 0% to 98%; and, from 13.4 to 17 min, solvent B was maintained at 98%.

Ionization was performed in both positive and negative electrospray ionization (ESI) modes. The mass spectrometry operating parameters in positive ion mode were as follows: spray voltage, 3500 V; sheath gas flow rate, 40 Arb; auxiliary gas flow rate, 10 Arb; purge gas flow rate, 1 Arb; ion transfer temperature, 275 °C; and heating temperature, 320 °C. The full scan settings were as follows: resolution, 60,000; scanning range, 70–1050 Da; RF lens, 70%. For data-dependent MS^2^ (ddMS^2^) analysis, the top N value was set to 5, with a resolution of 15,000 and the scanning range mode set to auto. The operating parameters in negative ion mode were as follows: spray voltage, 2800 V; sheath gas flow rate, 40 Arb; auxiliary gas flow rate, 10 Arb; purge gas flow rate, 1 Arb; ion transfer temperature, 275 °C; and heating temperature, 320 °C. The full scan settings were identical to those used in positive ion mode: resolution, 60,000; scanning range, 70–1050 Da; and RF lens, 70%. For ddMS^2^ analysis, the top N value was set to 5, with a resolution of 15,000 and the scanning range mode set to auto.

### 3.9. Liquid Chromatography/Mass Spectrometry

LC/MS analyses of F2 and F3 were performed with the same reagents and instrument parameters in Section 3.8. Chromatographic separation was performed on an HSS T3 column (1.8 μm, 100 mm × 2.1 mm; Waters, Milford, MA, USA). The liquid chromatography conditions in positive ion mode were as follows: column temperature, 40 °C, and injection volume, 2 μL. The mobile phase consisted of solvent A (0.1% formic acid in acetonitrile) and solvent B (0.1% formic acid in aqueous solution). The column was eluted at a flow rate of 0.25 mL/min, following the gradient program: the initial conditions started at 98% solvent B, held for 0–1 min; from 1 to 9 min, solvent B was reduced from 98% to 50%; from 9 to 12 min, solvent B decreased from 50% to 2%; from 12 to 13.5 min, solvent B was maintained at 2%; from 13.5 to 14 min, solvent B increased from 2% to 98%; and, from 14 to 20 min, solvent B was maintained at 98%. The liquid chromatography conditions in negative ion mode were as follows: column temperature, 40 °C, and injection volume, 2 μL. The mobile phase comprised solvent A (acetonitrile solution) and solvent B (5 mM of ammonium formate in aqueous solution). The column was eluted at a flow rate of 0.25 mL/min, following the gradient program: the initial conditions started at 98% solvent B, held for 0–1 min; from 1 to 9 min, solvent B was reduced from 98% to 50%; from 9 to 12 min, solvent B decreased from 50% to 2%; from 12 to 13.5 min, solvent B was maintained at 2%; from 13.5 to 14 min, solvent B increased from 2% to 98%; and, from 14 to 17 min, solvent B was maintained at 98%.

The mass spectrometry operating parameters in positive ion mode were as follows: spray voltage, 3500 V; sheath gas flow rate, 40 Arb; auxiliary gas flow rate, 10 Arb; ion transfer temperature, 325 °C; and heating temperature, 350 °C. The full scan settings were as follows: resolution, 60,000; scanning range, 100–1500 Da; RF lens, 70%. For data-dependent MS^2^ (ddMS^2^) analysis, the top N value was set to 5, with a resolution of 15,000 and the scanning range mode set to auto. The operating parameters in negative ion mode were as follows: spray voltage, 2500 V; sheath gas flow rate, 40 Arb; auxiliary gas flow rate, 10 Arb; ion transfer temperature, 325 °C; and heating temperature, 350 °C. The full scan settings were identical to those used in positive ion mode: resolution, 60,000; scanning range, 70–1050 Da; and RF lens, 70%. For ddMS^2^ analysis, the top N value was set to 5, with a resolution of 15,000 and the scanning range mode set to auto.

### 3.10. Statistical Analysis

The measurements were performed in triplicate. Statistical analysis was performed using SPSS 19.0 software (IBM Corporation, Armonk, NY, USA, 2010), and the results were expressed as the mean ± standard deviation. The data were analyzed using one-way ANOVA, followed by the S-N-K test, with a significance level of *p* < 0.05. Origin 2021 software (OriginLab Corporation, Northampton, MA, USA, 2021) and SIMCA 14.1 software were used.

## 4. Conclusions

Silkworm is a widely accepted hypoglycemic food in Asia, and *B. batryticatus* is a dried silkworm body, which is derived by the infection (or artificial inoculation) of *B. bassiana*. Nowadays, *B. batryticatus* is widely used in clinical practice for antiepilepsy, anticonvulsion, antidiabetes and blood lipid regulation, but there is still a lack of functional compound data. In this study, *B. batryticatus* was prepared, extracted, and separated using solvents of different polarities. Compared with the extracts of healthy silkworm larvae, the total reducing power, hydroxyl radical scavenging capacity, superoxide anion radical scavenging capacity, α-glucosidase inhibitory activity of the methanol extract of *B. batryticatus* demonstrated comprehensive superiority. Non-targeted LC-MS was further used to compare the differences in characteristic metabolites between *B. batryticatus* and healthy silkworm larvae. The results showed that fatty acids and their derivatives in *B. batryticatus* extract were significantly increased. Furthermore, the *B. batryticatus* extract was fractionated and separated according to polarity using solvents of different polarities, and the chloroform fraction of *B. batryticatus* exhibited the strongest α-glucosidase inhibitory activity. LC-MS was used to identify the compound composition of chloroform fraction. The results showed that nitrogen-containing heterocyclic compounds and fatty acid derivatives may contribute to the strong α-glucosidase inhibitory ability of chloroform fraction. The results provide a theoretical basis for the clinical application of *B. batryticatus*, and the active molecules in chloroform fraction offer potential for the development of hypoglycemic drugs. However, the hypoglycemic ability and mechanism of specific active substances in chloroform fraction of *B. batryticatus* still require further experiments such as molecular dynamic simulations or in vivo experiments of specific active substances.

## Figures and Tables

**Table 1:** Significant metabolite differences between *B. batryticatus* and healthy larvae samples in positive and negative ion modes. Compounds selected by OPLS-DA and VIP analysis (VIP > 1.5, *p* < 0.05) and univariate statistics (log_2_ FC > 1 or <−1, *p* < 0.05).

**NO**	**RT** **(min)**	Proposed Compound	Molecular Formula	Mass Error (ppm)	Theoretical Exact Mass (*m/z*)	Assigned Adduct	*p*-Values	Log_2_ (FC)	VIP Score
1	0.7	Ornithine	C_5_H_12_N_2_O_2_	0.11	132.08989	[M + H]^+^	5.19 × 10^−5^	−1.27	0.26
2	0.78	Carnitine	C_7_H_15_NO_3_	−0.01	161.10519	[M + H]^+^	1.30 × 10^−4^	1.95	0.88
3	0.78	α,α-Trehalose	C_12_H_22_O_11_	−4.06	342.11482	[M − H]^−^	3.71 × 10^−4^	−2.42	0.50
4	0.79	Methionine sulfoxide	C_5_H_11_NO_3_S	0.21	165.04600	[M + H]^+^	5.50 × 10^−6^	−5.53	0.36
5	0.79	2-Hydroxymethylpiperidine-3,4,5-triol	C_6_H_13_NO_4_	−0.53	163.08437	[M − H]^−^	5.21 × 10^−4^	4.28	0.58
6	0.79	Trigonelline	C_7_H_7_NO_2_	−0.01	137.04768	[M + H]^+^	1.08 × 10^−4^	2.19	0.43
7	0.81	Serine	C_3_H_7_NO_3_	0.23	105.04262	[M + CAN + H]^+^	1.77 × 10^−2^	−1.75	0.44
8	0.85	4-Nitrobenzoic acid	C_7_H_5_NO_4_	−2.55	167.02143	[M − H]^−^	1.24 × 10^−3^	5.14	0.65
9	0.88	Nicotinic acid	C_6_H_5_NO_2_	−0.23	123.03200	[M + H]^+^	9.98 × 10^−4^	−2.15	0.50
10	0.9	Arginine	C_6_H_14_N_4_O_2_	0.21	174.11171	[M + H]^+^	3.22 × 10^−02^	−2.63	0.48
11	1.11	7-Methylguanine	C_6_H_7_N_5_O	0.91	165.06521	[M + H]^+^	6.99 × 10^−4^	3.02	0.35
12	1.11	Acetyl-carnitine	C_9_H_17_NO_4_	0.38	203.11584	[M + H]^+^	1.05 × 10^−4^	3.03	0.58
13	1.17	Prolylleucine	C_11_H_20_N_2_O_3_	0.34	228.14747	[M + H]^+^	6.18×10^−4^	1.79	0.45
14	1.19	3-Methyladenine	C_6_H_7_N_5_	0.01	149.07015	[M + H]^+^	3.39 × 10^−4^	4.06	0.33
15	1.2	Acrylic acid	C_3_H_4_O_2_	−3.00	72.02091	[M − H]^−^	6.63 × 10^−6^	1.73	0.52
16	1.27	3-Butene-1,2,3-tricarboxylic acid	C_7_H_8_O_6_	−2.26	188.03166	[M − H]^−^	1.59 × 10^−6^	3.24	1.22
17	1.29	Proline	C_5_H_9_NO_2_	−0.93	115.06322	[M + H]^+^	5.07 × 10^−4^	1.56	0.50
18	1.33	2′-Deoxyadenosine	C_10_H_13_N_5_O_3_	0.14	251.10187	[M + H]^+^	2.93 × 10^−4^	−3.22	0.38
19	1.42	3-Carboxy-4-hydroxyphenyl-β-D-glucopyranoside	C_13_H_16_O_9_	−1.51	316.07895	[M − H]^−^	4.65 × 10^−3^	−4.47	0.59
20	1.45	Isoleucine	C_6_H_13_NO_2_	−0.67	131.09454	[M + H]^+^	9.85 × 10^−4^	−2.52	4.57
21	1.51	Gallic acid	C_7_H_6_O_5_	−2.65	170.02107	[M − H]^−^	1.48 × 10^−4^	3.57	0.84
22	1.58	Citric acid	C_6_H_8_O_7_	−2.28	192.02656	[M − H]^−^	1.24 × 10^−2^	1.39	2.23
23	1.84	Propionylcarnitine	C_10_H_19_NO_4_	0.01	217.13141	[M + H]^+^	1.96 × 10^−5^	3.34	0.37
24	2	Glutaric acid	C_5_H_8_O_4_	−1.44	132.04207	[M + H − H_2_O]^+^	3.02 × 10^−5^	−1.77	0.28
25	2.08	3-Hydroxy-3-(methoxycarbonyl) pentanedioic acid	C_7_H_10_O_7_	−2.35	206.04217	[M − H]^−^	9.86 × 10^−3^	2.71	0.43
26	2.39	N6-Me-Adenosine	C_11_H_15_N_5_O_4_	0.51	281.11255	[M + H]^+^	1.06 × 10^−4^	−2.19	0.16
27	2.4	Kynurenine	C_10_H_12_N_2_O_3_	0.88	208.08498	[M + H]^+^	6.57 × 10^−4^	−4.28	0.51
28	2.44	Unknown 1	C_18_H_26_O_11_	−2.07	418.14665	[M + FA − H]^−^	5.47 × 10^−4^	−1.68	0.42
29	2.45	Methylimidazoleacetic acid	C_6_H_8_N_2_O_2_	0.13	140.05860	[M + H]^+^	2.99 × 10^−2^	4.23	0.34
30	2.52	Methylsuccinic acid	C_5_H_8_O_4_	−2.01	132.04199	[M − H]^−^	4.54 × 10^−2^	1.12	0.80
31	2.65	Unknown 2	C_11_H_15_N_5_O_3_S	−0.35	297.08946	[M + H]^+^	1.34 × 10^−6^	3.58	0.50
32	2.66	Adipic acid	C_6_H_10_O_4_	−1.69	146.05766	[M − H]^−^	8.98 × 10^−6^	1.75	0.75
33	2.69	Unknown 3	C_17_H_34_N_4_O_4_	−0.26	358.25791	[M + 2H]^+^	1.57 × 10^−4^	4.4	0.22
34	2.79	Unknown 4	C_18_H_36_N_4_O_4_	−0.32	372.27354	[M + 2H]^+^	1.70 × 10^−6^	3.93	0.31
35	2.8	Sorbic acid	C_6_H_8_O_2_	−0.88	112.05233	[M + H]^+^	1.60 × 10^−4^	2.59	0.29
36	2.83	3-Hydroxybenzoic acid	C_7_H_6_O_3_	−1.95	138.03142	[M − H]^−^	5.32 × 10^−3^	−2.58	1.10
37	2.85	2-Naphthylamine	C_10_H_9_N	−1.62	143.07327	[M + H]^+^	1.06 × 10^−3^	−2.81	0.15
38	2.86	7-hydroxy-6-methoxy-2H-chromen-2-one	C_10_H_8_O_4_	−0.04	192.04225	[M + H]^+^	1.48 × 10^−6^	4.45	0.21
39	2.87	3-(2,5-Dihydroxyphenyl)-2-propenoic acid	C_9_H_8_O_4_	−2.21	180.04186	[M − H]^−^	5.10 × 10^−4^	−2.76	1.10
40	2.88	3-Hydroxy-3,5,5-trimethyl-4-(3-oxo-1-buten-1-ylidene) cyclohexyl β-D-glucopyranoside	C_19_H_30_O_8_	−0.17	386.19400	[M + H]^+^	2.93 × 10^−5^	−4.91	0.30
41	2.92	Unknown 5	C_15_H_22_O_5_	−1.87	282.14620	[M − H]^−^	7.21 × 10^−3^	1.25	0.28
42	2.97	Tryptophan	C_11_H_12_N_2_O_2_	−2.06	204.08946	[M − H]^−^	4.44 × 10^−4^	2.28	0.32
43	3	4-Acetyl-4-phenylpiperidine	C_13_H_17_NO	0.29	203.13107	[M + H]^+^	1.35 × 10^−4^	4.33	1.11
44	3.01	N-Acetyl-leucine	C_8_H_15_NO_3_	−1.62	173.10491	[M − H]^−^	4.77 × 10^−3^	−1.82	0.57
45	3.02	7,7-dimethyl-3-spiro (4,4,-dimethyl-2,6-dioxocyclohexyl)-1,2,3,4,5,6,7,8-octahydro-5-quinolinone	C_18_H_25_NO_3_	0.25	303.18352	[M + H]^+^	4.22 × 10^−4^	3.13	0.19
46	3.02	Unknown 6	C_18_H_30_O_10_	−2.88	406.18273	[M − H]^−^	1.02 × 10^−7^	−2.50	0.53
47	3.04	Trifolin	C_21_H_20_O_11_	−1.75	448.09978	[M − H]^−^	6.33 × 10^−8^	−1.06	0.69
48	3.05	4-Hydroxybenzaldehyde	C_7_H_6_O_2_	−1.89	122.03655	[M − H]^−^	3.83 × 10^−4^	−1.44	0.64
49	3.06	4-[4-(4-Hydroxy-3-methoxyphenyl) tetrahydro-1H,3H-furo [3,4-c] furan-1-yl]-2-methoxyphenyl hexopyranoside	C_26_H_32_O_11_	−1.78	520.19353	[M + FA − H]^−^	1.34 × 10^−3^	−5.27	0.85
50	3.06	Suberic acid	C_8_H_14_O_4_	−2.34	174.08880	[M − H]^−^	2.77 × 10^−6^	−2.46	1.60
51	3.1	2-(acetylamino)-3-(1H-indol-3-yl) propanoic acid	C_13_H_14_N_2_O_3_	−1.78	246.10000	[M − H]^−^	3.65 × 10^−4^	3.69	0.34
52	3.1	Phenylalanine	C_9_H_11_NO_2_	−0.33	165.07892	[M + H]^+^	3.58 × 10^−3^	−3.24	0.34
53	3.25	3-amino-2-phenyl-2H-pyrazolo [4,3-c] pyridine-4,6-diol	C_12_H_10_N_4_O_2_	0.16	242.08042	[M + H]^+^	4.78 × 10^−6^	5.55	0.68
54	3.33	2-(6-Hydroxyhexyl)-3-methylenesuccinic acid	C_11_H_18_O_5_	−2	230.11496	[M − H]^−^	8.89 × 10^−6^	−4.27	0.64
55	3.42	Indole-3-acetic acid	C_10_H_9_NO_2_	−0.45	175.06325	[M + H]^+^	1.11 × 10^−3^	−1.28	0.14
56	3.5	1-(5-chloro-2-methoxyphenyl)-3-phenyl-2,5-dihydro-1H-pyrrole-2,5-dione	C_17_H_12_ClNO_3_	−0.04	313.05056	[M + NH_4_]^+^	2.44 × 10^−4^	5.19	0.13
57	3.63	7-methoxy-1-methyl-3H,4H,9H-pyrido [3,4-b] indole	C_13_H_14_N_2_O	0.12	214.11064	[M + H]^+^	9.60 × 10^−7^	−6.69	0.22
58	3.63	3-Hydroxy-Caprylic acid	C_8_H_16_O_3_	−2.33	160.10957	[M − H]^−^	1.38 × 10^−3^	−1.59	0.26
59	3.63	Phenmetrazine	C_11_H_15_NO	−0.4	177.11529	[M + H]^+^	1.70 × 10^−4^	3.44	0.39
60	3.66	5-[4-(3-hydroxy-4-methoxyphenyl)-hexahydrofuro [3,4-c] furan-1-yl]-2-methoxyphenol	C_20_H_22_O_6_	−0.2	358.14157	[M + H − H_2_O]^+^	3.52 × 10^−4^	3.27	0.16
61	3.84	Corchorifatty acid F	C_18_H_32_O_5_	−1.89	328.22435	[M − H]^−^	1.36 × 10^−6^	−1.09	3.20
62	3.92	6-(7-methyloctyl)-1H,3H,4H,6H-furo [3,4-c] furan-1-one	C_15_H_24_O_3_	−0.04	252.17254	[M + H − H_2_O]^+^	1.42 × 10^−4^	−1.24	0.19
63	4.63	6,8-Dihydroxy-9,12-octadecadienoic acid	C_18_H_32_O_4_	−1.58	312.22957	[M − H]^−^	2.29 × 10^−4^	−1.26	0.61
64	5	Unknown 7	C_13_H_20_O_3_	0.21	224.14129	[M + H]^+^	3.48 × 10^−6^	−2.29	0.18
65	5.79	13-Hydroxy-9,11-octadecadienoic acid	C_18_H_30_O_3_	−2.25	294.21883	[M − H]^−^	8.12 × 10^−8^	−1.74	2.20
66	5.79	1-Linoleoyl glycerol	C_21_H_38_O_4_	−1.13	354.27661	[M + H − H_2_O]^+^	3.60 × 10^−3^	3.23	0.19
67	6.23	Docosapentaenoic acid	C_22_H_34_O_2_	0.19	330.25594	[M + H]^+^	8.59 × 10^−4^	−4.37	0.16
68	6.39	Sphingosine (d18:1)	C_18_H_37_NO_2_	−0.1	299.28240	[M + H]^+^	1.19 × 10^−3^	4.57	0.23
69	6.41	10-Hydroxy-2-decenoic acid	C_10_H_18_O_3_	−3.37	186.12497	[M − H]^−^	1.79 × 10^−4^	5.57	0.63
70	6.42	17α-Methyl-androstan-3-hydroxyimine-17β-ol	C_20_H_33_NO_2_	−4.67	319.24964	[M + H]^+^	1.01 × 10^−2^	3.12	0.33
71	6.66	12(13)-DiHOME	C_18_H_34_O_4_	−1	314.24540	[M − H]^−^	1.80 × 10^−5^	−1.86	0.56
72	6.73	Arachidonic acid	C_20_H_32_O_2_	0.38	304.24035	[M + H]^+^	3.03 × 10^−4^	−5.26	0.22
73	6.86	Ergothioneine	C_9_H_15_N_3_O_2_S	−0.02	229.08849	[M + H]^+^	3.51 × 10^−4^	5.77	0.25
74	6.92	3-Hydroxy myristic acid	C_14_H_28_O_3_	−2.67	244.20319	[M − H]^−^	4.99 × 10^−4^	3.14	1.34
75	6.99	5-[-5-(Hydroxymethyl)-1,2,4a-trimethyl-1,2,3,4,4a,7,8,8a-octahydro-1-naphthalenyl]-3-methylpentanoic acid	C_20_H_34_O_3_	−2.24	322.25007	[M − H]^−^	1.87 × 10^−3^	−1.48	0.59
76	7.19	6-Hydroxy-4-Octadecenoic Acid	C_18_H_34_O_3_	−2.5	298.25005	[M − H]^−^	7.17 × 10^−3^	−1.34	3.21
77	7.32	9,11-Conjugated linoleic acid	C_18_H_32_O_2_	−2.44	280.23955	[M − H]^−^	9.30 × 10^−6^	1.47	0.54
78	7.39	2-Hydroxymyristic acid	C_14_H_28_O_3_	−2.45	244.20325	[M − H]^−^	5.00 × 10^−3^	5.96	0.86
79	7.46	Labdanolic acid	C_20_H_36_O_3_	−2.04	324.26578	[M − H]^−^	5.17 × 10^−4^	−2.78	0.83
80	7.53	Ethyl palmitoleate	C_18_H_34_O_2_	−0.63	282.25570	[M + H]^+^	2.21 × 10^−3^	6.84	2.98
81	7.53	Oleoyl ethanolamide	C_20_H_39_NO_2_	−0.05	325.29806	[M + H]^+^	2.77 × 10^−3^	−4.39	0.67
82	8.03	Dihomo-γ-Linolenoyl Ethanolamide	C_22_H_39_NO_2_	0.02	349.29809	[M + H]^+^	6.48 × 10^−4^	−4.51	0.51
83	8.09	Unknown 8	C_20_H_35_NO	−0.27	305.27178	[M + H]^+^	1.25 × 10^−3^	−6.69	1.01
84	8.19	Eicosapentaenoic acid	C_20_H_30_O_2_	−2.19	302.22392	[M − H]^−^	4.53 × 10^−4^	−2.55	1.62
85	8.19	Linolenic acid ethyl ester	C_20_H_34_O_2_	−0.51	306.25573	[M + H]^+^	1.55 × 10^−4^	3.66	0.19
86	8.22	Palmitoyl ethanolamide	C_18_H_37_NO_2_	−0.57	299.28226	[M + H]^+^	6.96 × 10^−4^	−3.32	3.17
87	8.25	9-Hydroxy-10,11-octadecadienoic acid	C_18_H_32_O_3_	−2.8	296.23432	[M − H]^−^	4.96 × 10^−5^	−1.47	0.87
88	8.54	Tomatidine	C_27_H_45_NO_2_	−0.47	415.34483	[M + H]^+^	3.21 × 10^−3^	4.82	0.27
89	8.85	Palmitoleic acid	C_16_H_30_O_2_	−2.54	254.22393	[M − H]^−^	4.29 × 10^−4^	−2.02	0.35
90	8.88	Oleamide	C_18_H_35_NO	−0.21	281.27181	[M + H]^+^	8.79 × 10^−7^	1.05	5.08
91	8.92	Octadecenoic acid methyl ester	C_19_H_36_O_2_	0.06	296.27155	[M + H]^+^	3.04 × 10^−3^	4.83	0.67
92	9.25	Stearoyl ethanolamide	C_20_H_41_NO_2_	−0.41	327.31359	[M + H]^+^	4.00 × 10^−4^	−5.13	2.51
93	9.5	12-Oxo phytodienoic acid	C_18_H_28_O_3_	−0.41	292.20372	[M + H]^+^	7.96 × 10^−6^	−1.2	0.20
94	10.02	Cholecalciferol	C_27_H_44_O	0	384.33922	[M + H − H_2_O]^+^	1.46 × 10^−5^	−2.07	0.36
95	10.05	10-hydroxy-2,4a,6a,6b,9,9,12a-heptamethyl-13-oxo-1,2,3,4,4a,5,6,6a,6b,7,8,8a,9,10,11,12,12a,12b,13,14b-icosahydropicene-2-carboxylic acid	C_30_H_46_O_4_	−4.03	470.33771	[M + H]^+^	1.92 × 10^−5^	3.81	0.18
96	10.19	Unknown 9	C_30_H_48_O_2_	−0.37	440.36527	[M + H]^+^	2.77 × 10^−3^	−2.29	0.17
97	10.41	Maslinic acid	C_30_H_48_O_4_	−0.3	472.35512	[M + NH_4_]^+^	2.08 × 10^−4^	4.73	0.22
98	10.46	Methanandamide	C_23_H_39_NO_2_	−0.09	361.29805	[M + H]^+^	1.06 × 10^−3^	−3.28	0.14
99	12.13	17-Hydroxykauran-19-oic acid	C_20_H_32_O_3_	−0.18	320.23509	[M + H]^+^	1.20 × 10^−3^	3.59	0.42
100	12.43	Octadecatrienoic acid methyl ester	C_19_H_32_O_2_	−0.08	292.24021	[M + H]^+^	8.35 × 10^−3^	1.69	0.09
101	12.56	Cholest-4-en-3-one	C_27_H_44_O	−0.51	384.33902	[M + H]^+^	7.52 × 10^−5^	−2.02	0.94

*p*-Values obtained from analysis of *t*-test using a *p*-value threshold <0.05 for significant differences. Log fold change (Log_2_ FC) values were calculated to compare the healthy larvae and *B. batryticatus* samples.

**Table 2:** The top 20 compounds based on their concentrations in the chloroform fraction (F2).

Classification	UHPLC-MS			
	Compound Name	RT (min)	*m/z*	Mode +/−
Amino acids, amino acid analogs and amino acid derivatives	Proline (F2-1)	1.06	116.07	+
	Threo-3-Phenylserine (F2-2)	8.27	182.08	+
Fatty acids and fatty acid esters	Chorifatty acid F (F2-3)	9.10	327.22	−
	9,12,13-Trihydroxy-15-octadecenoic acid (F2-4)	9.54	329.23	−
	9,10-Dihydroxy-12-octadecenoic acid (F2-5)	12.15	313.24	−
	HOTrE (F2-6)	12.78	293.21	−
	HpODE (F2-7)	13.32	311.22	−
	1,2-dihydroxyheptadec-16-yn-4-yl acetate (F2-8)	14.28	349.23	−
Phenolic and flavonoid compounds	Salicylic acid (F2-9)	2.53	137.02	−
	α-Cyano-3-hydroxycinnamic acid (F2-10)	7.8 0	188.04	−
	Quercetin (F2-11)	9.55	301.04	−
Amide compounds	Stearamide (F2-12)	14.19	284.29	+
	Oleamide (F2-13)	15.50	282.28	+
Ketone compounds	3-(1-Hydroxyethyl)-2,3,6,7,8,8a-hexahydropyrrolo [1,2-a] pyrazine-1,4-dione (F2-14)	4.34	199.11	+
	7-Hydroxy-6-methoxy-2H-chromen-2-one (F2-15)	6.74	193.05	+
	4-Hydroxy-6-[2-(2-methyl-1,2,4a,5,6,7,8,8a-octahydronaphthalen-1-yl) ethyl] oxan-2-one (F2-16)	13.21	275.20	+
	1-(2,3-dihydro-2,3-dimethyl-1H-pyrrolo [3,4-b] quinolin-1-yl)-3,5-dimethyl-2,6-dimethoxyphenyl)-3-(2,4-dimethoxyphenyl) propan-1-one (F2-17)	13.92	611.29	+
	1′-Ethylspiro [6,7-dihydro-2H-furo [2,3-f] indole-3,4′-piperidine]-5-yl)-[4-[2-methyl-4-(5-methyl-1,3,4-oxadiazol-2-yl) phenyl] phenyl] methanone (F2-18)	14.54	535.27	+
Other compounds	Senkyunolide H (F2-19)	9.34	247.09	+
	Unknown (C_18_H_32_O_4_) (F2-20)	12.27	335.22	+

## Data Availability

All data supporting reported results can be found within this manuscript and Supplementary Materials.

## Supplementary Materials

The following supporting information can be downloaded at https://www.mdpi.com/article/10.3390/molecules30051021/s1, Table S1: Chemical constituents in the chloroform fraction concentrate (F2) of *B. batryticatus* extract as identified by LC-MS.

## References

[1] Hoang T.N.N., Nguyen Q.L., Le T.N.N., Vo N.H., Dong T.A.D., Le T.H.A. (2024). Comparative Study on the Hypoglycemic Effects of Different Parts of Musa balbisiana. Food Sci. Nutr..

[2] Soory M. (2009). Relevance of nutritional antioxidants in metabolic syndrome, ageing and cancer: Potential for therapeutic targeting. Curr. Drug Targets Infect. Disord..

[3] Abdel-Daim M.M., El-Tawil O.S., Bungau S.G., Atanasov A.G. (2019). Applications of antioxidants in metabolic disorders and degenerative diseases: Mechanistic approach. Oxid. Med. Cell. Longev..

[4] Abdel-Daim M.M., Zakhary N.I., Aleya L., Bungǎu S.G., Bohara R.A., Siddiqi N.J. (2018). Aging, metabolic, and degenerative disorders: Biomedical value of antioxidants. Oxid. Med. Cell. Longev..

[5] Ighodaro O.M., Akinloye O.A. (2017). Anti-diabetic potential of *Sapium ellipticum* (Hochst) Pax leaf extract in Streptozotocin (STZ)-induced diabetic Wistar rats. BMC Complement. Altern. Med..

[6] Alfarisi H., Sa’diah S., Wresdiyati T. (2020). Polyphenol Profile, Antioxidant and Hypoglycemic Activity of *Acalypha hispida* Leaf Extract. Indian J. Pharm. Sci..

[7] Elbashir S.M.I., Devkota H.P., Wada M., Kishimoto N., Moriuchi M., Shuto T., Misumi S., Kai H., Watanabe T. (2018). Free radical scavenging, α-glucosidase inhibitory and lipase inhibitory activities of eighteen Sudanese medicinal plants. BMC Complement Altern. Med..

[8] Uysal S., Zengin G., Aktumsek A., Karatas S. (2015). Fatty acid composition, total sugar content and anti-diabetic activity of methanol and water extracts of nine different fruit tree leaves collected from Mediterranean region of Turkey. Int. J. Food Prop..

[9] Pares R.B., Alves L.F.A. (2016). Controle e prevenção da calcinose branca em *Bombyx mori* L. (Lepidoptera: Bombycidae). Arq. Do Inst. Biológico.

[10] Rattana S., Katisart T., Butiman C., Sungthong B. (2019). Total flavonoids, total phenolics, 1-deoxynojirimycin content and alpha-glucosidase inhibitory activity of Thai silkworm races (*Bombyx mori* Linn.). Pak. J. Pharm. Sci..

[11] Ryu K.-S., Lee H.-S., Kim K.-Y., Kim M.-J., Sung G.-B., Ji S.-D., Kang P.-D. (2013). 1-deoxynojirimycin content and blood glucose-lowering effect of silkworm (*Bombyx mori*) extract powder. Int. J. Indust. Entomol..

[12] Mahanta D.K., Komal J., Samal I., Bhoi T.K., Dubey V.K., Pradhan K., Nekkanti A., Gouda M.N.R., Saini V., Negi N. (2023). Nutritional aspects and dietary benefits of “Silkworms”: Current scenario and future outlook. Front. Nutr..

[13] Xing D., Shen G., Li Q., Xiao Y., Yang Q., Xia Q. (2019). Quality formation mechanism of stiff silkworm, *Bombyx batryticatus* using UPLC-Q-TOF-MS-based metabolomics. Molecules.

[14] Lin L., Zhang Y., Li Y., Fu H., Hu J., Zhou Y., Xu Y., Xia G., Sun X., Yang H. (2020). Identification of signature proteins of processed *Bombyx batryticatus* by comparative proteomic analysis. Int. J. Biol. Macromol..

[15] Hu M., Yu Z., Wang J., Fan W., Liu Y., Li J., Xiao H., Li Y., Peng W., Wu C. (2017). Traditional Uses, Origins, Chemistry and Pharmacology of *Bombyx batryticatus*: A Review. Molecules.

[16] Lim H.-S., Kim J.-S., Moon B.C., Ryu S.M., Lee J., Park G. (2019). *Batryticatus Bombyx* protects dopaminergic neurons against MPTP-induced neurotoxicity by inhibiting oxidative damage. Antioxidants.

[17] Kumar V., Tewari S.K., Awasthi A.K. (1994). Surface ultrastructure of *Beauveria bassiana* infecting silkworm *Bombyx mori* Linn. Curr. Sci..

[18] Choi B.H., Ji S.D., Son J.G., Nguyen P., Kim K.Y., Park Y.H., Koh Y.H. (2017). Phytochemicals and silk proteins in mature silkworm powders responsible for extended life expectancy and enhanced resistances to Parkinson’s disease. J. Asia. Pac. Entomol..

[19] Wannee S., Luchai B. (2020). 1-Deoxynojirimycin and polyphenolic composition and antioxidant activity of different native Thai silkworm (*Bombyx mori*) larvae. J. King Saud Univ. Sci..

[20] Paul D., Dey S. (2014). Essential amino acids, lipid profile and fat-soluble vitamins of the edible silkworm *Bombyx mori* (Lepidoptera: Bombycidae). Int. J. Trop. Insect Sci..

[21] Ali M.F.Z., Nakahara S., Otsu Y., Ido A., Miura C., Miura T. (2021). Effects of functional polysaccharide from silkworm as an immunostimulant on transcriptional profiling and disease resistance in fish. J. Insects Food Feed.

[22] Cermeño M., Bascón C., Amigo-Benavent M., Felix M., FitzGerald R.J. (2022). Identification of peptides from edible silkworm pupae (*Bombyx mori*) protein hydrolysates with antioxidant activity. J. Funct. Foods.

[23] Bae S.-M., Jo Y.-Y., Lee K.-G., Kim H.-B., Kweon H. (2016). Antioxidant activity of silkworm powder treated with protease. Int. J. Indust. Entomol..

[24] Ávila-Hernández J.G., Carrillo M.L., Cruz R.D.I., Wong-Paz J.E. (2020). *Beauveria bassiana* secondary metabolites: A review inside their production systems, biosynthesis, and bioactivities. Mex. J. Biotechnol..

[25] Das U.N. (2018). Arachidonic acid in health and disease with focus on hypertension and diabetes mellitus: A review. J. Adv. Res..

[26] Khadem S., Marles R.J. (2010). Monocyclic phenolic acids; hydroxy-and polyhy-droxybenzoic acids: Occurrence and recent bioactivity studies. Molecules.

[27] Lesjak M., Beara I., Simin N., Pintać D., Majkić T., Bekvalac K., Orčić D., Mimica-Dukić N. (2018). Antioxidant and anti-inflammatory activities of quercetin and its derivatives. J. Funct. Foods.

[28] Strugała P., Tronina T., Huszcza E., Gabrielska J. (2017). Bioactivity in vitro of quercetin glycoside obtained in *Beauveria bassiana* culture and its interaction with liposome membranes. Molecules.

[29] Koirala N., Thuan N.H., Ghimire G.P., Van Thang D., Sohng J.K. (2016). Methylation of flavonoids: Chemical structures, bioactivities, progress and perspectives for biotechnological production. Enzyme Microb. Technol..

[30] Abdel-Daim M.M., Abo-EL-Sooud K., Aleya L., Bungǎu S.G., Najda A., Saluja R. (2018). Alleviation of drugs and chemicals toxicity: Biomedical value of antioxidants. Oxid. Med. Cell. Longev..

[31] Ramírez G., Zavala M., Pérez J., Zamilpa A. (2012). In vitro screening of medicinal plants used in Mexico as antidiabetics with glucosidase and lipase inhibitory activities. Evid. Based. Complement. Alternat. Med..

[32] Hasnat H., Shompa S.A., Islam M.M., Alam S., Richi F.T., Emon N.U., Ashrafi S., Ahmed N.U., Chowdhury M.N.R., Fatema N. (2024). Flavonoids: A treasure house of prospective pharmacological potentials. Heliyon.

[33] Xiao J. (2017). Dietary flavonoid aglycones and their glycosides: Which show better biological significance?. Crit. Rev. Food Sci. Nutr..

[34] Liu S.H., Liu Q., Lei L., Sun S.J., Li C.Y., Huan Y., Hou S.C., Shen Z.F. (2020). The Chinese patent medicine, Jin-tang-ning, ameliorates hyperglycemia through improving β cell function in pre-diabetic KKAy mice. Chin. J. Nat. Med..

[35] Hong K.S., Yun S.M., Cho J.M. (2018). Silkworm (*Bombyx mori*) powder supplementation alleviates alcoholic fatty liver disease in rats. J. Funct. Foods.

[36] Amobonye A., Bhagwat P., Pandey A., Singh S., Pillai S. (2020). Biotechnological potential of *Beauveria bassiana* as a source of novel biocatalysts and metabolites. Crit. Rev. Biotechnol..

[37] Patocka J. (2016). Bioactive metabolites of entomopathogenic fungi *Beauveria bassiana*. Mil. Med. Sci. Lett..

[38] Vesa C.M., Bungau S.G. (2023). Novel molecules in diabetes mellitus, dyslipidemia and cardiovascular disease. Int. J. Mol. Sci..

[39] Han L., Zhang M., Li F., Su J., Wang R., Li G., Yang X. (2023). 10-hydroxy-2-decenoic acid alleviates lipopolysaccharide-induced intestinal mucosal injury through anti-inflammatory, antioxidant, and gut microbiota modulation activities in chickens. Front. Microbiol..

[40] Gaspar A., Martins M., Silva P., Garrido E.M., Garrido J., Firuzi O., Miri R., Saso L., Borges F. (2010). Dietary phenolic acids and derivatives. Evaluation of the antioxidant activity of sinapic acid and its alkyl esters. J. Agric. Food Chem..

[41] Sepand M.R., Razavi-Azarkhiavi K., Omidi A., Zirak M.R., Sabzevari S., Kazemi A.R., Sabzevari O. (2016). Effect of acetyl-L-carnitine on antioxidant status, lipid peroxidation, and oxidative damage of arsenic in rat. Biol. Trace Elem. Res..

[42] Mani K., Vitenberg T., Khatib S., Opatovsky I. (2023). Effect of entomopathogenic fungus *Beauveria bassiana* on the growth characteristics and metabolism of black soldier fly larvae. Pestic. Biochem. Physiol..

[43] Richard D., Kefi K., Barbe U., Bausero P., Visioli F. (2008). Polyunsaturated fatty acids as antioxidants. Pharmacol. Res..

[44] Su C.H., Hsu C.H., Ng L.T. (2013). Inhibitory potential of fatty acids on key enzymes related to type 2 diabetes. Biofactors.

[45] Malki F., Touati A., Hamza K., Moulay S., Baltas M. (2016). Antioxidant activity of a series of amides. J. Mater. Environ. Sci..

[46] Fowler C.J., Jonsson K.O., Tiger G. (2001). Fatty acid amide hydrolase: Biochemistry, pharmacology, and therapeutic possibilities for an enzyme hydrolyzing anandamide, 2-arachidonoylglycerol, palmitoylethanolamide, and oleamide. Biochem. Pharmacol..

[47] Tanvir R., Javeed A., Rehman Y. (2018). Fatty acids and their amide derivatives from endophytes: New therapeutic possibilities from a hidden source. FEMS Microbiol. Lett..

[48] Xu Y.J., Luo F., Gao Q., Shang Y., Wang C. (2015). Metabolomics reveals insect metabolic responses associated with fungal infection. Anal. Bioanal. Chem..

[49] Huang L., Cheng T., Xu P., Cheng D., Fang T., Xia Q. (2009). A genome-wide survey for host response of silkworm, *Bombyx mori* during pathogen *Bacillus bombyseptieus* infection. PLoS ONE.

[50] Liu J., Zhang D., Zhu Y., Wang Y., He S., Zhang T. (2018). Enhancing the in vitro Antioxidant Capacities via the interaction of amino acids. Emir. J. Food Agric..

[51] Ko Y.-C., Choi H.S., Kim J.-H., Kim S.-L., Yun B.-S., Lee D.-S. (2020). Coriolic Acid (13-(S)-Hydroxy-9Z, 11E-octadecadienoic Acid) from Glasswort (*Salicornia herbacea* L.) Suppresses Breast Cancer Stem Cell through the Regulation of c-Myc. Molecules.

[52] Sharma E., Shruti P.S., Singh S., Singh T., Kaur P., Jodha B., Srivastava Y., Munshi A., Singh S. (2023). Trehalose and its diverse biological potential. Curr. Protein Pept. Sci..

[53] Olomola T.O., Mphahlele M.J., Gildenhuys S. (2020). Benzofuran-selenadiazole hybrids as novel α-glucosidase and cyclooxygenase-2 inhibitors with antioxidant and cytotoxic properties. Bioorg. Chem..

[54] Altowyan M.S., Barakat A., Al-Majid A.M., Al-Ghulikah H.A. (2019). Spiroindolone analogues as potential hypoglycemic with dual inhibitory activity on α-amylase and α-glucosidase. Molecules.

[55] Tafesse T.B., Bule M.H., Khoobi M., Faramarzi M.A., Abdollahi M., Amini M. (2020). Coumarin-based scaffold as α-glucosidase inhibitory activity: Implication for the development of potent antidiabetic agents. Mini Rev. Med. Chem..

[56] Dehnavi F., Alizadeh S.R., Ebrahimzadeh M.A. (2021). Pyrrolopyrazine derivatives: Synthetic approaches and biological activities. Med. Chem. Res..

[57] Mingoia F., Di Sano C., D’Anna C., Fazzari M., Minafra L., Bono A., Monica G.L., Martorana A., Almerico A., Lauria A. (2023). Synthesis of new antiproliferative 1, 3, 4-substituted-pyrrolo [3, 2-c] quinoline derivatives, biological and in silico insights. Eur. J. Med. Chem..

[58] Laddha A.P., Kulkarni Y.A., Chen H., Zhang M. (2021). Nitrogenous Compounds from Plant Origin in Management of Diabetes Mellitus. Structure and Health Effects of Natural Products on Diabetes Mellitus.

[59] Liu H., Li Z., Xia X., Zhang R., Wang W., Xiang X. (2023). Chemical profile of phenolic extracts from rapeseed meal and inhibitory effects on α-glucosidase: UPLC-MS/MS analysis, multispectral approaches, molecular simulation and ADMET analysis. Food Res. Int..

[60] Malunga L.N., Joseph Thandapilly S., Ames N. (2018). Cereal-derived phenolic acids and intestinal alpha glucosidase activity inhibition: Structural activity relationship. J. Food Biochem..

[61] Huang Y., Wu Y., Yin H., Du L., Chen C. (2023). Senkyunolide I: A review of its phytochemistry, pharmacology, pharmacokinetics, and drug-likeness. Molecules.

[62] Fan Y., Wang J., Jian J., Wen Y., Li J., Tian H., Crommen J., Jiang Z., Bi W., Zhang T. (2024). High-throughput discovery of highly selective reversible hMAO-B inhibitors based on at-line nanofractionation. Acta Pharm. Sin. B.

[63] Zhang Q., Yang Y.X., Li S.Y., Wang Y.L., Yang F.Q., Chen H., Xia Z.N. (2017). An ultrafiltration and high performance liquid chromatography coupled with diode array detector and mass spectrometry approach for screening and characterizing thrombin inhibitors from Rhizoma Chuanxiong. J. Chromatogr. B.

[64] Keivani N., Piccolo V., Marzocchi A., Maisto M., Tenore G.C., Summa V. (2024). Optimization and validation of procyanidins extraction and phytochemical profiling of seven herbal matrices of nutraceutical interest. Antioxidants.

[65] Tang J., Zhang W., Yuan R., Shu Y., Liu G., Zheng B., Tu J. (2024). Fortification of yogurt with mulberry leaf extract: Effects on physicochemical, antioxidant, microbiological and sensory properties during 21-days of storage. Heliyon.

[66] Jiang W., Zheng S., Yuan C., Gao Q., Xiang C., Tian S., Zhao Y. (2023). Study on extraction technology and antioxidant activity of total alkaloids from *Hemsleya chinensis* based on orthogonal design and BP neural network. Heliyon.

[67] Tu J., Liu G., Jin Y., Tang C., Yao T., Zhuo J., Li Q., Liu L., Wang J. (2022). Enrichment of γ-aminobutyric acid in mulberry leaves and the inhibitory effects of the water extract on ACE and α-glucosidase activity. Ind. Crops Prod..

[68] Aloo S.O., Ofosu F.K., Muchiri M.N., Vijayalakshmi S., Pyo C.-G., Oh D.-H. (2023). In Vitro Bioactivities of Commonly Consumed Cereal, Vegetable, and Legume Seeds as Related to Their Bioactive Components: An Untargeted Metabolomics Approach Using UHPLC–QTOF-MS2. Antioxidants.

