# Supplementary material for: Solvent Fractionation and LC-MS Profiling, Antioxidant Properties, and α-Glucosidase Inhibitory Activity of Bombyx batryticatus
Source: Molecules. 2025 Feb 23;30(5):1021. doi: 10.3390/molecules30051021 (PMC11901607; doi:10.3390/molecules30051021)
Supplement: Supplementary file 1 [file molecules-30-01021-s001.zip › molecules-3476952-supplementary.pdf]

Table S1. Chemical constituents in the chloroform fraction concentrate (F2) of *B. batryticatus* extract as identified by LC-MS.

No	RT (min)	Proposed compound	Formula	m/z	Reference Ion
1	0.846	Malic acid	C4 H6 O5	133.014	[M-H]-1
2	0.864	N6, N6, N6-Trimethyl-L-lysine	C9 H20 N2 O2	189.16	[M+H]+1
3	0.901	1-DNJ	C6 H13 N O4	164.092	[M+H]+1
4	0.907	Arginine	C6 H14 N4 O2	175.119	[M+H]+1
5	0.922	Serine	C3 H7 N O3	106.05	[M+H]+1
6	0.936	Aspartic acid	C4 H7 N O4	134.045	[M+H]+1
7	0.941	UDP-N-acetylglucosamine	C17 H27 N3 O17 P2	606.074	[M-H]-1
8	0.96	Carnitine	C7 H15 N O3	162.113	[M+H]+1
9	0.968	Citrulline	C6 H13 N3 O3	176.103	[M+H]+1
10	0.97	2-Aminobutyric acid	C4 H9 N O2	104.071	[M+H]+1
11	0.974	Iditol	C6 H14 O6	183.086	[M+H]+1
12	0.988	Glucose	C6 H12 O6	161.046	[M-H-H2O]-1
13	1.01	α, α-Trehalose	C12 H22 O11	387.114	[M+FA-H]-1
14	1.027	N3, N4-Dimethyl-arginine	C8 H18 N4 O2	203.15	[M+H]+1
15	1.056	Proline	C5 H9 N O2	116.071	[M+H]+1
16	1.076	Unknown 12	C6 H7 N O2	126.055	[M+H]+1
17	1.082	Adenosine	C10 H13 N5 O4	268.104	[M+H]+1
18	1.358	4-Hydroxybutyric acid (GHB)	C4 H8 O3	103.04	[M-H]-1
19	1.62	Acetylarginine	C8 H16 N4 O3	217.129	[M+H]+1
20	1.885	Gentisic acid	C7 H6 O4	153.019	[M-H]-1
21	1.928	Unknown 13	C11 H13 N O4	222.077	[M-H]-1
22	2.253	Salicylic acid	C7 H6 O3	137.025	[M-H]-1
23	2.534	6-Hydroxypicolinic acid	C6 H5 N O3	138.02	[M-H]-1
24	2.608	Unknown 14	C8 H8 O4	167.035	[M-H]-1
25	3.302	Unknown 15	C9 H8 O4	179.035	[M-H]-1
26	3.445	Corey lactone	C8 H12 O4	155.07	[M+H-H2O]+1
27	4.335	3-(1-Hydroxyethyl)-2,3,6,7,8,8a-hexahydropyrrolo[1,2-a] pyrazine-1,4-dione	C9 H14 N2 O3	199.108	[M+H]+1
28	4.468	2'-O-Methyladenosine	C11 H15 N5 O4	282.12	[M+H]+1
29	4.666	2,3-Dihydro-1-benzofuran-2-carboxylic acid	C9 H8 O3	163.04	[M-H]-1
30	4.676	N6-Me-Adenosine	C11 H15 N5 O4	282.12	[M+H]+1
31	4.786	Unknown 16	C15 H22 O5	281.14	[M-H]-1

32	4.851	Zeatin	C10 H13 N5 O	220.119	[M+H]+1
33	4.88	2'-Deoxyadenosine	C10 H13 N5 O3	252.109	[M+H]+1
34	4.994	3-Hydroxyphenylacetic acid	C8 H8 O3	151.04	[M-H]-1
35	5.193	2'-Deoxyinosine	C10 H12 N4 O4	253.093	[M+H]+1
36	5.581	Prolylleucine	C11 H20 N2 O3	229.155	[M+H]+1
37	5.876	2-(Acetylamino)-3-(1H-indol-3-yl) propanoic acid	C13 H14 N2 O3	245.093	[M-H]-1
38	5.957	5-(6-hydroxy-6-methyloctyl)-2,5-dihydrofuran-2-one	C13 H22 O3	209.154	[M+H-H2O]+1
39	6.002	N-Cyclopropyl-2-[(2S,3R,4S,5R)-3,4-dihydroxy-5-{[(methylsulfonyl) amino] methyl} tetrahydro-2-furanyl] acetamide	C11 H20 N2 O6 S	307.097	[M-H]-1
40	6.096	(2E)-5-hydroxy-N-[3-(5-{3-[(2E)-5-hydroxy-3-methylpent-2-enamido] propyl}-3,6-dioxopiperazin-2-yl) propyl] -3-methylpent-2-enamide	C22 H36 N4 O6	453.271	[M+H]+1
41	6.13	3-[(4-Hydroxyphenyl) methyl]-octahydropyrrolo[1,2-a] pyrazine-1,4-dione	C14 H16 N2 O3	261.123	[M+H]+1
42	6.271	Citral	C10 H16 O	153.127	[M+H]+1
43	6.271	Unknown 17	C10 H16 N2 O2	197.128	[M+H]+1
44	6.409	2-(2-Amino-3-methylbutanamido)-3-phenylpropanoic acid	C14 H20 N2 O3	265.155	[M+H]+1
45	6.572	10-HDA	C10 H18 O3	187.133	[M+H]+1
46	6.615	4-(4-Hydroxy-2,6,6-trimethyl-3-{[3,4,5-trihydroxy-6-(hydroxymethyl) oxan-2-yl] oxy} cyclohex-1-en-1-yl) butan-2-one	C19 H32 O8	411.199	[M+Na]+1
47	6.658	3-Acetamidophenol	C8 H9 N O2	150.056	[M-H]-1
48	6.685	3-Hydroxy-3,5,5-trimethyl-4-(3-oxo-1-buten-1-ylidene) cyclohexyl β-D-glucopyranoside	C19 H30 O8	387.201	[M+H]+1
49	6.727	Indole-3-carboxylic acid	C9 H7 N O2	160.041	[M-H]-1
50	6.738	7-Hydroxy-6-methoxy-2H-chromen-2-one	C10 H8 O4	193.05	[M+H]+1
51	6.805	N-(2-hydroxyphenyl) acetamide	C8 H9 N O2	152.071	[M+H]+1
52	6.863	Unknown 18	C15 H22 O3	251.164	[M+H]+1
53	6.92	Unknown 19	C15 H24 O2	237.185	[M+H]+1
54	7.09	4-Methyl-5-oxo-2-pentyl-2,5-dihydrofuran-3-carboxylic acid	C11 H16 O4	213.112	[M+H]+1
55	7.149	5-[4-(3-Hydroxy-4-methoxyphenyl)-hexahydrofuro[3,4-c] furan-1-yl]-2-methoxyphenol	C20 H22 O6	341.138	[M+H-H2O]+1
56	7.256	Unknown 20	C11 H18 N2 O2	211.144	[M+H]+1

57	7.299	Quercetin 3-O-rhamnoside-7-O-glucoside	C27 H30 O16	611.161	[M+H]+1
58	7.43	tert-Butyl N-[1-(aminocarbonyl)-3-methylbutyl] carbamate	C11 H22 N2 O3	231.17	[M+H]+1
59	7.533	Cyclo(leucylprolyl)	C11 H18 N2 O2	211.144	[M+H]+1
60	7.559	Quercetin-3β-D-glucoside	C21 H20 O12	465.103	[M+H]+1
61	7.707	Isoferulic acid	C10 H10 O4	195.065	[M+H]+1
62	7.743	Unknown 21	C12 H20 O4	251.125	[M+Na]+1
63	7.795	α-Cyano-3-hydroxycinnamic acid	C10 H7 N O3	188.035	[M-H]-1
64	7.822	2-(acetylamino)-3-phenylpropanoic acid	C11 H13 N O3	208.097	[M+H]+1
65	7.861	4-[4-(4-Hydroxy-3-methoxyphenyl) tetrahydro-1H,3H-furo[3,4-c] furan-1-yl]-2-methoxyphenyl hexopyranoside	C26 H32 O11	519.187	[M-H]-1
66	7.898	Syringaresinol	C22 H26 O8	417.156	[M-H]-1
67	7.926	Trifolin	C21 H20 O11	447.093	[M-H]-1
68	7.933	Cyclo(phenylalanyl-prolyl)	C14 H16 N2 O2	245.128	[M+H]+1
69	8.02	1b,2-Dimethyl-7a-(prop-1-en-2-yl)-1aH,1bH,2H,3H,4H,5H,7H,7aH-naphtho[1,2-b] oxirene-3,7-diol	C15 H22 O3	251.164	[M+H]+1
70	8.128	Indole-3-lactic acid	C11 H11 N O3	206.081	[M+H]+1
71	8.268	Threo-3-Phenylserine	C9 H11 N O3	182.081	[M+H]+1
72	8.304	1-[4-Hydroxy-3-(3-methylbut-2-en-1-yl) phenyl] ethan-1-one	C13 H16 O2	205.122	[M+H]+1
73	8.352	4-Methoxy-6-(prop-2-en-1-yl)-2H-1,3-benzodioxole	C11 H12 O3	175.075	[M+H-H2O]+1
74	8.386	10-Nitrooleate	C18 H33 N O4	328.248	[M+H]+1
75	8.613	9-Nitrooleate	C18 H33 N O4	328.248	[M+H]+1
76	8.661	4-Oxododecanedioic acid	C12 H20 O5	267.12	[M+Na]+1
77	8.834	Unknown 22	C13 H22 O5	241.144	[M+H-H2O]+1
78	9.049	10-Nitrolinoleate	C18 H31 N O4	326.232	[M+H]+1
79	9.097	Corchorifatty acid F	C18 H32 O5	327.218	[M-H]-1
80	9.104	1,4-Dihydroxy-1,4-dimethyl-7-(propan-2-ylidene)-decahydroazulen-6-one	C15 H24 O3	253.18	[M+H]+1
81	9.343	Senkyunolide H	C12 H16 O4	247.094	[M+Na]+1
82	9.47	5,10,15-Trimethyl-4,9,13-trioxatetracyclo[10.3.0.0^{1,5}.0^{8,10}]pentadec-1(15)-en-14-one	C15 H20 O4	247.133	[M+H-H2O]+1
83	9.542	9,12,13-Trihydroxy-15-octadecenoic acid	C18 H34 O5	329.234	[M-H]-1
84	9.551	Quercetin	C15 H10 O7	301.035	[M-H]-1
85	9.613	C75	C14 H22 O4	237.148	[M+H-H2O]+1

86	9.708	Dihydrokawain	C14 H16 O3	233.117	[M+H]+1
87	9.814	Ergothioneine	C9 H15 N3 O2 S	230.097	[M+H]+1
88	9.985	Unknown 23	C12 H18 O4	227.128	[M+H]+1
89	10.037	Unknown 24	C16 H24 O3	265.18	[M+H]+1
90	10.264	Ageratriol	C15 H24 O3	235.169	[M+H-H2O]+1
91	10.294	Unknown 25	C12 H20 O4	229.144	[M+H]+1
92	10.445	Unknown 26	C18 H30 O5	327.217	[M+H]+1
93	10.455	6-(7-Methyloctyl)-1H,3H,4H,6H-furo[3,4-c] furan-1-one	C15 H24 O3	235.169	[M+H-H2O]+1
94	10.509	Unknown 27	C22 H32 N2 O2	357.254	[M+H]+1
95	10.543	N-(1-Benzyl-4-piperidinyl)-5-(tert-butyl)-2-methyl-3-furamide	C22 H30 N2 O2	355.238	[M+H]+1
96	10.907	α-Linolenic acid	C18 H30 O2	279.232	[M+H]+1
97	11.17	5(6)-EET Ethanolamide	C22 H37 N O3	346.274	[M+H-H2O]+1
98	11.22	9,13-12-Oxophytodienoic acid	C18 H28 O3	293.211	[M+H]+1
99	11.468	2,3-Dinor-11β-prostaglandin F2α	C18 H30 O5	349.2	[M+Na]+1
100	11.909	2-(8-Hydroxy-4a,8-dimethyl-decahydronaphthalen-2-yl) prop-2-enoic acid	C15 H24 O3	235.169	[M+H-H2O]+1
101	12.059	12(13)-DiHOME	C18 H34 O4	313.239	[M-H]-1
102	12.102	9,10,13-Trihydroxyoctadeca-11,15-dienoic acid	C18 H32 O5	351.214	[M+Na]+1
103	12.146	9,10-Dihydroxy-12-octadecenoic acid	C18 H34 O4	313.239	[M-H]-1
104	12.153	11(12)-DiHET	C20 H34 O4	321.242	[M+H-H2O]+1
105	12.269	Unknown 28	C18 H32 O4	335.219	[M+Na]+1
106	12.297	Glycerophospho-N-palmitoyl ethanolamine	C21 H44 N O7 P	452.279	[M-H]-1
107	12.412	9(10)-DiHOME	C18 H34 O4	313.239	[M-H]-1
108	12.541	2-(3,8-Dimethyl-2-oxo-1,2,4,5,6,7,8,8a-octahydroazulen-5-yl) prop-2-enoic acid	C15 H20 O3	249.149	[M+H]+1
109	12.587	3-Hydroxy myristic acid	C14 H28 O3	243.197	[M-H]-1
110	12.623	Bis(4-ethylbenzylidene) sorbitol	C24 H30 O6	437.193	[M+Na]+1
111	12.72	Unknown 29	C18 H34 O4	297.243	[M+H-H2O]+1
112	12.778	13-HOTrE	C18 H30 O3	293.212	[M-H]-1
113	12.823	4-Dodecylbenzenesulfonic acid	C18 H30 O3 S	325.184	[M-H]-1
114	12.848	Myristyl sulfate	C14 H30 O4 S	293.179	[M-H]-1

115	12.868	3-Aminonon-5-enoic acid	C9 H17 N O2	172.133	[M+H]+1
116	13.068	Oleoyl-α-lysophosphatidic acid	C21 H41 O7 P	435.252	[M-H]-1
117	13.212	4-Hydroxy-6-[2-(2-methyl-1,2,4a,5,6,7,8,8a-octahydronaphthalen-1-yl) ethyl] oxan-2-one	C18 H28 O3	275.201	[M+H-H2O]+1
118	13.316	9-HpODE	C18 H32 O4	311.223	[M-H]-1
119	13.439	Triphenyl phosphate	C18 H15 O4 P	327.078	[M+H]+1
120	13.523	Tributyl phosphate	C12 H27 O4 P	267.172	[M+H]+1
121	13.605	Sphingosine	C18 H37 N O2	282.279	[M+H-H2O]+1
122	13.63	N-Phenyl-2-naphthylamine	C16 H13 N	220.112	[M+H]+1
123	13.648	Thromoboxane B1	C20 H36 O6	395.24	[M+Na]+1
124	13.737	Erucamide	C22 H43 N O	338.342	[M+H]+1
125	13.815	9-Oxo-10,12-octadecadienoic acid	C18 H30 O3	336.253	[M+ACN+H]+1
126	13.848	2,4-Dihydroxyheptadec-16-en-1-yl acetate	C19 H36 O4	311.258	[M+H-H2O]+1
127	13.92	Manidipine	C35 H38 N4 O6	611.286	[M+H]+1
128	13.951	Dibutyl hexanedioate	C14 H26 O4	259.19	[M+H]+1
129	14.05	Mono(2-ethylhexyl) phthalate (MEHP)	C16 H22 O4	279.159	[M+H]+1
130	14.059	N-({5-[3-(3,4-Dimethoxyphenyl)-1-methyl-1H-pyrazol-5-yl]-1-azabicyclo [2.2.2] oct-2-yl} methyl)-2-ethylbutanamide	C26 H38 N4 O3	453.289	[M-H]-1
131	14.19	Stearamide	C18 H37 N O	284.295	[M+H]+1
132	14.24	Ethyl palmitoleate	C18 H34 O2	283.263	[M+H]+1
133	14.244	Tretinoin	C20 H28 O2	299.202	[M-H]-1
134	14.28	1,2-Dihydroxyheptadec-16-yn-4-yl acetate	C19 H34 O4	349.235	[M+Na]+1
135	14.282	5α-Dihydrotestosterone	C19 H30 O2	291.232	[M+H]+1
136	14.334	Linoleoyl ethanolamide	C20 H37 N O2	324.29	[M+H]+1
137	14.537	1'-Ethyl-6,7-dihydro-5H-spiro(furo(2,3-f) indole-3,4'-piperidin)-5-yl) (2')	C33 H34 N4 O3	535.27	[M+H]+1
138	14.785	Palmitoyl ethanolamide	C18 H37 N O2	300.29	[M+H]+1

139	14.964	Unknown 30	C40 H75 N O9	758.543	[M+FA-H]-1
140	15.008	Oleoyl ethanolamide	C20 H39 N O2	326.305	[M+H]+1
141	15.172	15-OxoEDE	C20 H34 O3	345.24	[M+Na]+1
142	15.5	Oleamide	C18 H35 N O	282.279	[M+H]+1
143	15.572	11,14,17-Eicosatrienoic acid	C20 H34 O2	305.249	[M-H]-1
